# Chemical capacitance measurements reveal the impact of oxygen vacancies on the charge curve of LiNi_0.5_Mn_1.5_O_4−*δ*_ thin films†

**DOI:** 10.1039/d3ta05086f

**Published:** 2023-10-16

**Authors:** Andreas E. Bumberger, Sergej Ražnjević, Zaoli Zhang, Gernot Friedbacher, Juergen Fleig

**Affiliations:** a Institute of Chemical Technologies and Analytics, TU Wien Vienna Austria andreas.bumberger@tuwien.ac.at; b Erich Schmid Institute for Materials Science Leoben Austria

## Abstract

The level of oxygen deficiency *δ* in high-voltage spinels of the composition LiNi_0.5_Mn_1.5_O_4−*δ*_ (LNMO) significantly influences the thermodynamic and kinetic properties of the material, ultimately affecting the cell performance of the corresponding lithium-ion batteries. This study presents a comprehensive defect chemical analysis of LNMO thin films with oxygen vacancy concentrations of 2.4% and 0.53%, focusing particularly on the oxygen vacancy regime around 4 V *versus* Li^+^/Li. A set of electrochemical properties is extracted from impedance measurements as a function of state-of-charge for the full tetrahedral-site regime (3.8 to 4.9 V *versus* Li^+^/Li). A defect chemical model (Brouwer diagram) is derived from the data, providing a coherent explanation for all important trends of the electrochemical properties and charge curve. Highly resolved chemical capacitance measurements allow a refining of the defect model for the oxygen vacancy regime, showing that a high level of oxygen deficiency not only impacts the amount of redox active Mn^3+/4+^, but also promotes the trapping of electrons in proximity to an oxygen vacancy. The resulting stabilisation of Mn^3+^ thereby mitigates the voltage reduction in the oxygen vacancy regime. These findings offer valuable insights into the complex influence of oxygen deficiency on the performance of lithium-ion batteries based on LNMO.

## Abstract 2

Chemical capacitance measurements reveal the impact of oxygen vacancies on the electrochemical properties of epitaxial LiNi_0.5_Mn_1.5_O_4−*δ*_ thin films. Oxygen deficiency not only impacts the amount of redox active Mn, but also promotes electron trapping.

## Graphical abstract

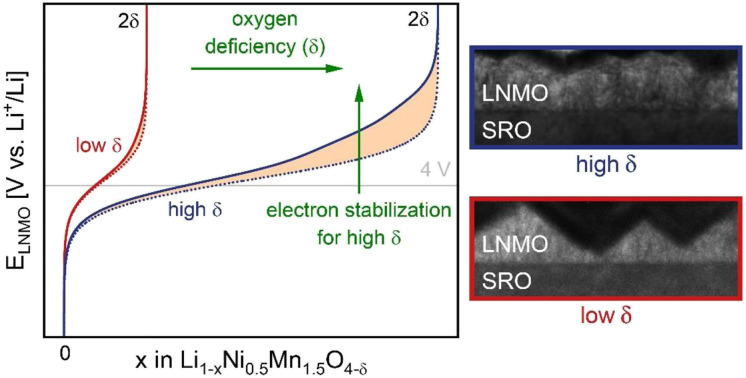

## Introduction

Over the past three decades, lithium-ion batteries (LIBs) have emerged as the key energy storage technology for a wide range of applications. In particular, the utilization of LIBs in electric vehicles requires not only high energy densities to ensure a sufficient driving range, but also high power densities to enable fast charging. The overall power density of a Li-ion cell strongly depends on the specific electrochemical properties of the electrode materials (*i.e.*, charge transfer resistance *R*_ct_, ionic conductivity *σ*_ion_, volume-specific chemical capacitance *C*^V^_chem_, Li chemical diffusion coefficient *D̃*). However, detailed studies dealing with the dependence of these properties on the state-of-charge (SOC) are rare. Interestingly, defect chemical models based on dilute-solution thermodynamics can offer valuable insights into the SOC-dependent transport properties of Li storage materials over a surprisingly wide range of charge carrier concentrations.^1–3^ Nonetheless, defect interactions inevitably come into play when exploiting the full charge/discharge capacity of a given Li storage material.

In the search for new cathode materials, the cobalt-free spinel of the nominal composition LiNi_0.5_Mn_1.5_O_4_ (LNMO) has been investigated as a promising candidate due to its potentially low cost, good rate capability and high voltage *versus* Li^+^/Li.^4–13^ In contrast to its isostructural parent material LiMn_2_O_4_ (LMO), where Mn is in the mixed valence state of +3.5, Mn in stoichiometric LiNi_0.5_Mn_1.5_O_4_ is fully oxidized to a valence state of +4, while Ni remains in the lower valence state +2. Upon charging, the extraction of Li from the 8a tetrahedral sites is therefore accompanied by the oxidation of Ni to a final valence state of +4 in Ni_0.5_Mn_1.5_O_4_. Thus, the high oxidation potential of Ni^2+/3+/4+^ is exploited to reach a voltage of approximately 4.7 V *versus* Li^+^/Li, compared to 4.0 V in the Mn^3+/4+^-based LMO.

However, stoichiometric LiNi_0.5_Mn_1.5_O_4_ is difficult to synthesize, due to the material's tendency towards oxygen deficiency, accompanied by partial reduction of Mn^4+^ to Mn^3+^.^6,10,11,14–20^ Due to the exothermal nature of the oxygen incorporation reaction, a high degree of oxidation is favoured by low temperatures and high oxygen partial pressures. Thus, the typical synthesis route towards stoichiometric LNMO ends with a slow cooling step under an oxygen-rich atmosphere to allow the material to equilibrate at the lowest possible temperature. It was shown that a high degree of oxidation in LNMO is closely related to Ni ordering on the octahedral sites. While high levels of oxygen deficiency favour the formation of the disordered phase with space group *Fd*3̄*m* (“d-LNMO”), samples close to the stoichiometric composition LiNi_0.5_Mn_1.5_O_4_ tend to crystallise in the ordered *P*4_3_32 phase (“o-LNMO”), although the difference between these two phases is hard to resolve by crystallographic experiments.^6^

From a defect chemical perspective, oxygen deficiency in LNMO can be realized either *via* oxygen vacancies according to LiNi_0.5_Mn_1.5_O_4−*δ*_ or *via* metal interstitials according to [LiNi_0.5_Mn_1.5_]_1+*δ*/(4−*δ*)_O_4_. However, these two cases are difficult to distinguish experimentally, because both configurations lead to a donor-doped material of the same chemical composition. Although the metal excess model has been discussed for both LMO and LNMO,^21–23^ the larger part of literature seems to adopt the viewpoint of oxygen nonstoichiometry,^6,17,20^ and in this paper we therefore also consider oxygen deficiency in terms of oxygen vacancies in LiNi_0.5_Mn_1.5_O_4−*δ*_. Charge neutrality then requires the reduction of 2*δ* formula units of Mn^4+^ to Mn^3+^, which can be expressed as LiNi_0.5_Mn^4+^_1.5−2*δ*_Mn^3+^_2*δ*_O_4−*δ*_. As a consequence, a fraction corresponding to 2δ × 100% of the total tetrahedral site capacity is moved from the 4.7 V (Ni^2+/3+/4+^) to the 4.0 V (Mn^3+/4+^) regime, resulting in a proportional loss of storable energy due to the lower average voltage.

However, the presence of oxygen vacancies does not simply move part of the total capacity from the high-voltage Ni- to the lower-voltage Mn-regime, but furthermore introduces a variety of possible defect interactions such as defect associates (*i.e.*, electronic and ionic trap states) that influence the material's SOC-dependent electrochemical properties in a nontrivial manner. Although computational studies have suggested a significant impact of such associates (trap states) on the voltage profile of LNMO electrodes,^17,24–26^ particularly in the oxygen vacancy regime around 4.0 V, there have been little efforts to quantitatively describe the experimentally observed shape of the LNMO charge curve as a function of oxygen vacancy concentration and defect interaction energies. Furthermore, although the overall cycling performances of ordered (low *δ*) and disordered (high *δ*) samples have often been compared, the precise variation of their electrochemical transport properties with *δ* and the state-of-charge is largely unknown.

In this work, we investigate the impact of oxygen vacancies on the electrochemical properties of epitaxial LNMO thin films by means of impedance measurements as a function of electrode potential. We report a full and detailed set of electrochemical properties (charge transfer resistance, ionic conductivity, chemical capacitance and chemical diffusion coefficient) as a function of SOC for two different levels of oxygen deficiency. Particularly the chemical capacitance (*i.e.*, differential capacity) turns out to be a highly valuable source of information regarding both ionic and electronic defect interactions in LNMO and their impact on the charge curve. For the first time, we provide a detailed defect chemical analysis of the charge curve in the oxygen vacancy regime around 4 V *versus* Li^+^/Li and show that the level of oxygen deficiency not only determines the amount of redox active Mn^3+/4+^ but furthermore impacts the defect interactions and hence the shape of the charge curve. More generally, the reported results pave the way towards a better understanding of the oxygen-nonstoichiometry in all Li-ion cathode materials and its effect on electrode performance.

## Results

### Epitaxial SRO/LNMO thin films

To analyse the effect of oxygen vacancies on the electrochemical properties of LNMO, two types of thin-film sample were prepared – one with a high and one with a low level of oxygen deficiency, as described in the experimental section. Although the post-oxidized sample is still weakly oxygen-deficient (see below), and its space group could not be unambiguously identified as *Fd*3̄*m* or *P*4_3_32, its structural and electrochemical properties are close to what is referred to as ordered LNMO in the literature, and we will therefore refer to the samples with high and low oxygen vacancy concentration as d-LNMO and o-LNMO (d = disordered, o = ordered), respectively.

**Fig. 1:**
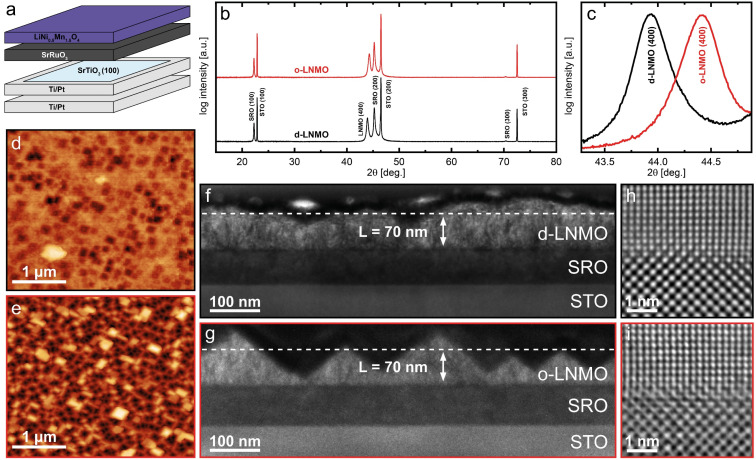
Structural characterisation of d-LNMO and o-LNMO thin film samples. (a) Schematic illustration showing the individual components of the overall sample. SRO and LNMO were deposited onto a (100)-oriented STO single crystal with Ti/Pt-coated edges. An additional layer of Ti/Pt was sputtered onto the backside for a better electrical contact. (b) Out-of-plane *θ*–2*θ* X-ray diffractogram showing only reflexes of the (*h*00) family for STO, SRO and LNMO, indicating epitaxial growth of both SRO and LNMO on STO. (c) Magnification of the LNMO (400) reflex from the X-ray diffractogram (b) around 2*θ* = 44°, clearly showing a decrease of the cubic lattice parameter from d-LNMO to o-LNMO. (d and e) AFM images of the samples surfaces of the d-LNMO and o-LNMO thin films, respectively. (f and g) Bright-field TEM images of the d-LNMO and o-LNMO samples, respectively, displaying a more defined pyramidal structure in the o-LNMO film. The estimated average thickness of 70 nm is indicated in both images. (h and i) ABS-filtered high-resolution TEM images of the SRO (bottom)/LNMO (top) interface of the d-LNMO and o-LNMO samples, confirming the epitaxial growth of LNMO on SRO.

The structural characterisation of typical d-LNMO and o-LNMO samples is summarised in Fig. 1. As shown in Fig. 1a, LNMO was deposited onto an epitaxial SrRuO_3_ (SRO) thin-film current collector on a (100)-oriented SrTiO_3_ (STO) single crystal substrate coated with Ti/Pt on its sides and backside. The *θ*–2*θ* X-ray diffractograms in Fig. 1b suggest that both o-LNMO and d-LNMO grow epitaxially on the (100)-oriented SRO thin film. For SRO and the STO substrate, only the (100), (200) and (300) reflexes are visible in the measured range of 2*θ*, with the SRO reflexes shifted to lower angles with respect to the substrate. For both samples, the SRO (200) reflex appears around 2*θ* = 45.2°, corresponding to an out-of-plane lattice parameter of approximately 4.01 Å. In a previous study,^2^ it was confirmed by reciprocal space mapping that SRO grows on STO as a compressively strained, epitaxial thin film, thereby adopting the in-plane lattice parameter of 3.91 Å from the substrate. As shown in Fig. 1c, the LNMO (400) reflex appears at 2*θ* = 43.9° and 2*θ* = 44.3° for d-LNMO and o-LNMO, corresponding to out-of-plane lattice parameters of 8.25 Å and 8.18 Å, respectively. This is in good agreement with literature, where a larger lattice parameter is reported for d-LNMO due to the larger ionic radius of Mn^3+^ compared to Mn^4+^.^18^

The epitaxial growth of both d- and o-LNMO on the SRO current collector is confirmed by the high-resolution TEM images in Fig. 1h and i, respectively. The close agreement of the measured out-of-plane lattice parameter with the bulk lattice parameters from literature, together with the large lattice mismatch of 5% between LNMO and SRO, suggests that, for both samples, the epitaxial LNMO film relaxes to its bulk in- and out-of-plane lattice parameter within a short distance from the interface, and does not adopt a significant amount of compressive strain from the substrate.

The AFM images of the d- and o-LNMO surfaces are shown in Fig. 1d and e, respectively, and show a clear difference in morphology between the disordered and ordered spinel samples. The d-LNMO film exhibits an ill-defined surface morphology with some octahedral imprints that hint at the characteristic morphology of (400)-oriented spinel thin films, an RMS roughness of 15 nm and a surface area that is 12% larger than the projected sample area. The o-LNMO film, on the other hand, exhibits a well-defined morphology with a clear preference for exposure of the (111) facets, resulting in an octahedrally imprinted structure with an RMS roughness of 45 nm and a surface area that is 59% larger than the projected area. The difference in morphology between d-LNMO and o-LNMO is even more evident in the corresponding bright-field TEM images in Fig. 1f and g, respectively, with the ordered sample showing clearly defined pyramids, while the disordered sample exhibits a smoother but more irregularly shaped surface.

For the d-LNMO thin film, an average thickness of 70 nm was estimated from the bright-field TEM images. For the o-LNMO film, the estimated average thickness in the cross section shown in Fig. 1g is only 56 nm. However, due to the strong thickness variation in the o-LNMO film, the comparatively small sample size of the TEM image and the fact that the same deposition rate and time were employed for both samples, we use the same average thickness (70 nm) for the data analysis of both d-LNMO and o-LNMO. Owing to the thickness variations, especially in the o-LNMO film, the geometry normalised properties deduced from the impedance spectra have to be regarded as averaged (effective) values. Although the presence of grain boundaries cannot be ruled out, our TEM investigations indicate a largely single-crystalline, dense thin film for both sample types. This is further supported by the Fourier transform (FT) images shown in Fig. S1 of the ESI† together with the corresponding HR-TEM images. For both samples, the FT patterns correspond to a spinel single crystal as viewed along the [010] zone axis with some additional amorphous contributions, which presumably stem from the amorphous surface layer introduced during FIB lamella preparation. For o-LNMO, some minor additional spots are observed, which could originate from the ordered *P*4_3_32 phase, or a small fraction of crystallites with different orientations. In either case, the films appear dense and largely single-crystalline, and we therefore consider the extracted electrochemical properties as representative also for the corresponding bulk material.

### DC measurements

**Fig. 2:**
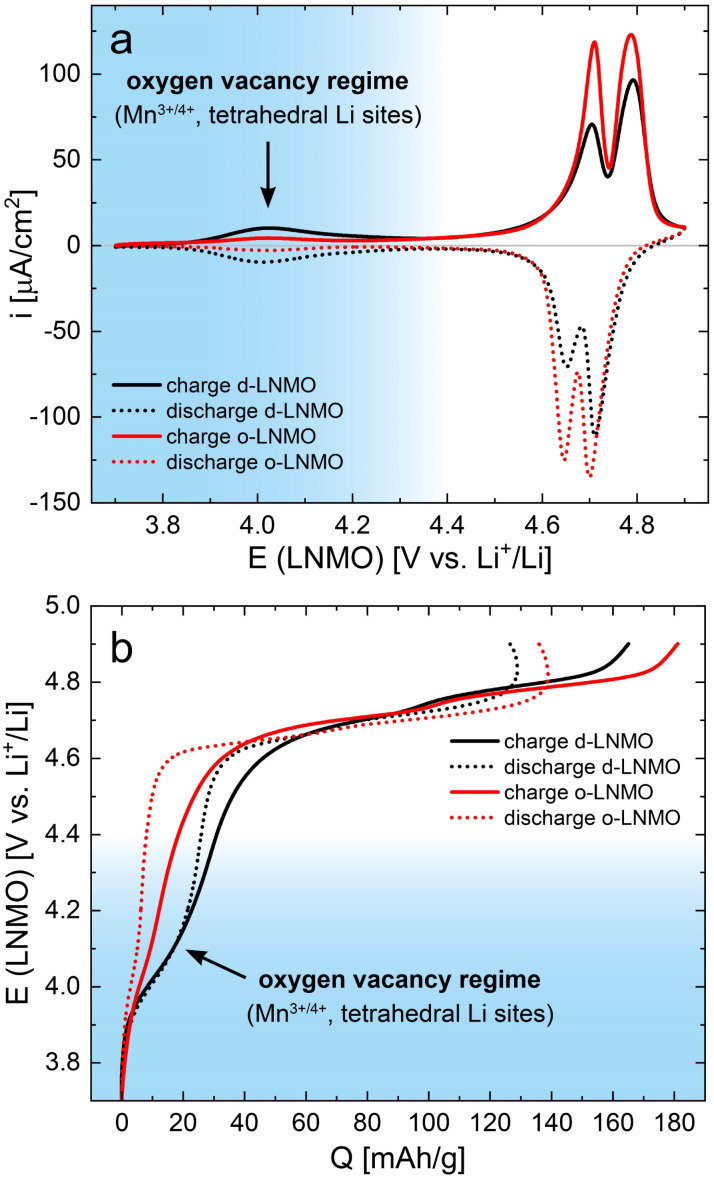
DC characterization *via* cyclic voltammetry of d-LNMO (black) and o-LNMO (red) samples prior to impedance measurements. (a) Cyclic voltammograms (fifth cycle, scan rate 1 mV s^−1^) showing clear differences between d-LNMO and o-LNMO in the 4.0 V and 4.7 V regimes. (b) Voltage *versus* charge profiles obtained *via* integration of the CV curves in (a). In both plots, the voltage range coloured in blue corresponds to the oxygen vacancy regime, which is the main focus of this study.

After cell assembly, the two different LNMO samples were characterized by cyclic voltammetry (CV) for 5 cycles at a scan rate of 1 mV s^−1^, to ensure sufficient electrochemical stability for the subsequent impedance measurements. The fifth cycle CV scans are plotted in Fig. 2a. Both samples exhibit the well-known double peak in current density around 4.7 V *versus* Li^+^/Li,^6,10,13^ corresponding to the removal/insertion of Li^+^ from the tetrahedral sites upon oxidation/reduction of Ni^2+/3+/4+^, with o-LNMO showing higher overall current densities for both the forward and the backward scan. While it may be tempting to assign these two peaks to the separate redox couples Ni^2+/3+^ and Ni^3+/4+^, the potential difference close to 100 mV between the two peaks is very similar to the double peak observed for LMO, where only one redox species (Mn^3+/4+^) is active, and the peak splitting has been reported to originate from Li ordering at the composition Li_0.5_Mn_2_O_4_.^27–31^ It is therefore likely, although experimentally not fully established, that a similar ordering occurs in LNMO and may either cause or at least contribute to the observed peak splitting.^10^

A second, broader and much lower current peak is observed around 4.0 V, which is significantly higher for d-LNMO than for o-LNMO. This peak can be assigned to the removal/extraction of Li^+^ from the tetrahedral sites upon oxidation/reduction of Mn^3+/4+^, stemming from the charge compensation of oxygen vacancies. Hence, this peak is the focus of our study. The much lower current densities observed for o-LNMO compared to d-LNMO in the 4.0 V region therefore confirms the successful, although still incomplete, incorporation of additional oxygen into the material in the post-annealing step under oxygen atmosphere.

For both samples, a significant amount of background current is observed, that increases up to approximately 10 μA cm^−2^ at 4.9 V. This background current presumably originates from side reactions due to either impurities or residual water content in the electrolyte or the onset of electrolyte oxidation at the highest voltages. Although the observed background currents are negligible compared to typical bulk electrode capacities in the mA h range, they are significant in the context of our thin film electrodes with capacities of only a few μA h. Accordingly, they have a noticeable impact on the coulombic efficiency, as discussed below. Cyclic voltammetry was therefore chosen over classical galvanostatic cycling, since the former allows for a better estimation of the background currents contributing to the measured charge/discharge capacities. A comparison of constant–current cycling data of d-LNMO and o-LNMO can be found, for example, in ref. 6, 11, and 16.

By integration, the CV curves in Fig. 2a can also be converted directly into potential *versus* charge curves, *i.e.*, voltage profiles, as shown in Fig. 2b. The integrated charge/discharge capacities amount to 165/127 mA h g^−1^ (d-LNMO) and 181/136 mA h g^−1^ (o-LNMO), with corresponding coulombic efficiencies of 77% and 75%, respectively. As discussed above, side reactions with the electrolyte, indicated by the oxidative background current in the CV scan, negatively impact the coulombic efficiency. Furthermore, these background currents lead to a distortion of the charge/discharge curves towards higher/lower capacities in the high-voltage region above 4.8 V. More generally, the voltage profiles appear particularly distorted in potential regions outside the main storage regime around 4.7 V, where the currents due to reversible Li intercalation/extraction are low and background currents are comparatively higher. Since these potential regions also correspond to stoichiometries with low charge carrier concentrations, where a dilute defect model could be applied to describe the material's electrochemical properties, the DC measurements shown in Fig. 2 do not provide sufficiently accurate data for a quantitative comparison with defect chemical calculations. For further investigations, we therefore resort to impedance measurements, as established in our previous studies on Li_1−*δ*_CoO_2_ and Li_2−*δ*_Mn_2_O_4_.^1,2^ This virtually eliminates the effect of background currents and allows us to extract not only the volume-specific chemical capacitance *C*^V^_chem_ (*i.e.*, differential capacity), but also the charge transfer resistance *R*_ct_, the ionic conductivity *σ*_ion_ and the Li chemical diffusion coefficient *D̃* as a function of SOC.

### Impedance measurements

**Fig. 3:**
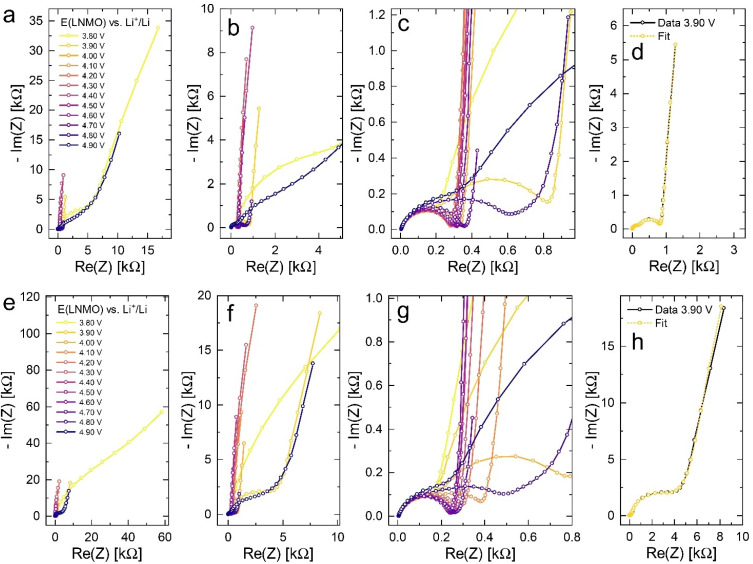
Impedance spectra of d-LNMO (a–d) and o-LNMO (e–h) as a function of electrode potential in the range of 3.80 V to 4.90 V *versus* Li^+^/Li in increments of 100 mV. For better overview, the remaining spectra in 10 mV increments are not shown. (a–c) Impedance spectra of d-LNMO at different magnifications. (d) Exemplary impedance fit for the d-LNMO spectrum at 3.90 V. (e–g) Impedance spectra of o-LNMO at different magnifications. (h) Exemplary impedance fit for the o-LNMO spectrum at 3.90 V.

Impedance spectra for LNMO electrode potentials of 3.80 to 4.90 V *versus* Li^+^/Li were measured in increments of 10 mV and are shown in Fig. 3 for both d-LNMO and o-LNMO in increments of 100 mV. For both samples, the frequency-dependent impedance response exhibits a similar variation with electrode potential, with the highest real and imaginary impedances at 3.80 and 4.90 V. The real impedance only shows a strong variation at low and high voltages and appears to remain relatively constant in the intermediate voltage range. The imaginary part of the impedance reaches a first minimum at 4.00 V and a second, more pronounced minimum around 4.70 V. The high-frequency region of the spectra, which is magnified in Fig. 3c (d-LNMO) and Fig. 3g (o-LNMO), consists of two semicircles, one of which exhibits a strong variation with electrode potential, whereas the other remains nearly constant throughout the entire set of measurements. At intermediate voltages, the two semicircles appear to be of similar magnitude and blend into each other, signifying a similar capacitance of the corresponding transport processes. The most notable difference between the d-LNMO and o-LNMO samples can be seen in the low and intermediate voltage region, up to approximately 4.40 V. For d-LNMO, the previously described variation of real and imaginary impedance around 4.00 V is more pronounced than for o-LNMO. For example, the impedance spectrum of o-LNMO at 3.90 V is very similar to the spectrum at 4.90 V, while for d-LNMO, the real and imaginary impedances are significantly lower at 3.90 V. Since both the real and imaginary parts of the electrode impedance are related to charge carrier concentrations in the material (see next section), this hints at a higher capacity of d-LNMO in the Mn-regime, as could already be estimated from the CV curves in Fig. 2a.

### Fitting of impedance spectra

**Fig. 4:**
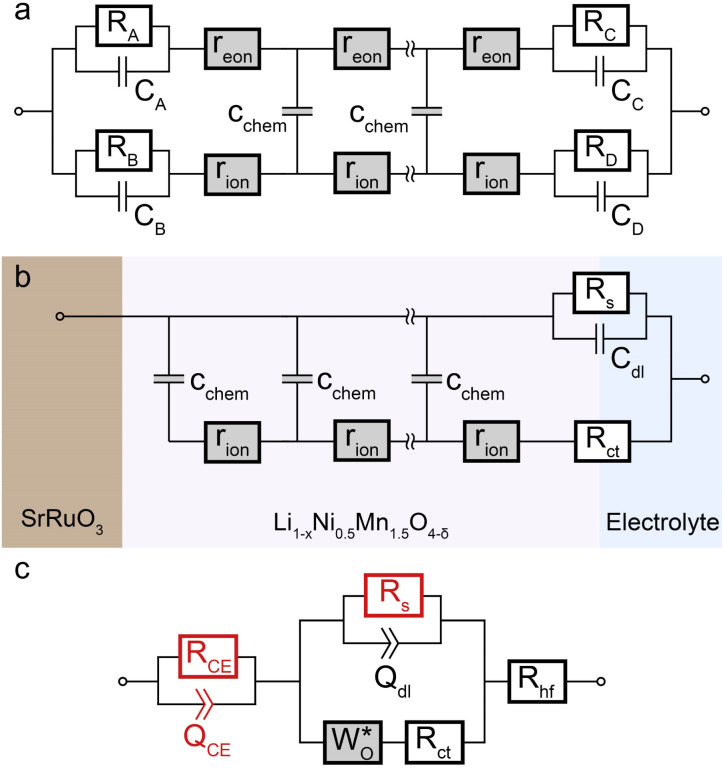
Stepwise derivation of the equivalent circuit used for impedance fitting. (a) General transmission line with four distinct R/C terminals. (b) Simplified transmission line obtained by (i) neglecting electronic resistances, (ii) assuming an ohmic electronic contact at the SRO/LNMO interface, and (iii) assuming an LNMO/electrolyte interface that allows for both Li^+^ and electron transfer. (c) Final equivalent circuit used for fitting, obtained by accounting for anomalous diffusion as well as for the contributions of the Li metal counter electrode (CE) and the electrolyte. Circuit elements with fixed parameters are marked in red, and their respective values are summarised in Table 1.

Impedance spectra of Li storage thin films have conventionally been fitted with Randles' circuit,^32,33^ which is based on an intuitive combination of an open Warburg element *W*_o_ with a charge transfer resistance *R*_ct_, a double-layer capacitance *C*_dl_ and a high-frequency offset resistance *R*_hf_. More generally, the impedance response of a Li storage electrode can be described by the general transmission line model proposed by Jamnik and Maier, shown in Fig. 4a. This physically derived impedance model considers the one-dimensional transport of mass and charge across a mixed ionic and electronic conductor (MIEC) slab of thickness *L* by two parallel resistive rails for ions and electrons, coupled by chemical capacitors, with *R*_eon_ = ∑*r*_eon_, *R*_ion_ = ∑*r*_ion_, and *C*_chem_ = ∑*c*_chem_.^34–38^ For a Li storage electrode, the volume-specific chemical capacitance can be defined as^39^1
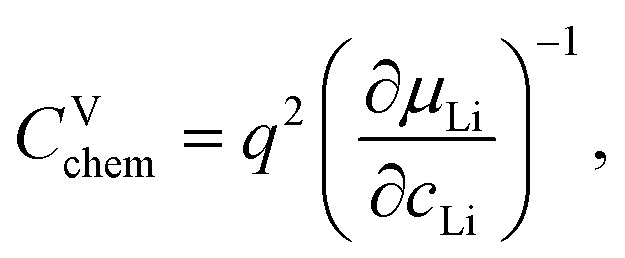
where *q* is the elementary charge, *c*_Li_ is the concentration of formally neutral Li. The Li chemical potential *μ*_Li_ of the MIEC *versus* Li metal is related to the Li activity *a*_Li_ in the MIEC *via*

2 *μ*_Li_ = *μ*_Li,metal_ + *kT* ln *a*_Li_,

with Boltzmann's constant *k* and the temperature *T*. The Li chemical potential and activity are both related to the electrode potential *E versus* Li metal *via*3
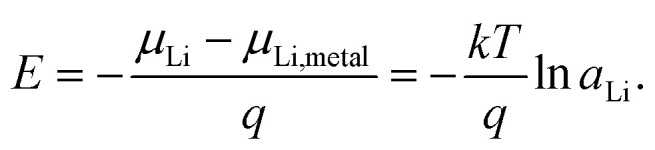
As in most other thin film impedance studies of LIB cathodes, we assume a comparatively high electronic conductivity *σ*_eon_ of the material such that *σ*_eon_ ≫ *σ*_ion_ and *R*_eon_ ≈ 0. Please note that if *σ*_ion_ and *σ*_eon_ were of similar magnitude, an SOC-dependent shift of the high-frequency offset would be expected, which is not observed experimentally. With a negligible electronic resistance, the chemical diffusion coefficient can be expressed as4
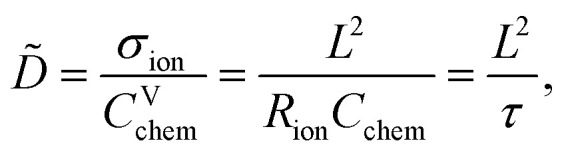
where *τ* is the time constant of chemical diffusion, that is, the Li storage process.^40^

For the present study, we further assume a current collector that presents an ohmic electronic contact to the mixed ionic and electronic conductor (LNMO), *i.e.*, a negligible contact resistance, and an LNMO/electrolyte interface that allows for both charge transfer (transport of Li^+^ across the interface) and side reactions (*i.e.*, electron transfer to or from the electrolyte, for example due to electrolyte oxidation at high voltages). The charge transfer and side reaction resistances are denoted as *R*_ct_ and *R*_s_, respectively. The electrochemical double-layer capacitance *C*_dl_ at the LNMO/electrolyte interface is placed on the electronic rail terminal to remain consistent with the commonly used Randles' circuit after replacement of the transmission line with an open Warburg element (see below). The resulting equivalent circuit is shown in Fig. 4b.

The remaining portion of the transmission line is identical to an ideal open Warburg element *W*_o_, which describes the impedance transition from semi-infinite to finite-space diffusion with characteristic phase angles of 45° and 90°, respectively. In our previous studies on Li_1−*δ*_CoO_2_ and Li_2−*δ*_Mn_2_O_4_,^1,2^ we have shown that the diffusional impedance of real intercalation electrodes can be fitted with an anomalous finite-space diffusion element 
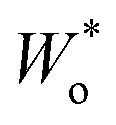
 embedded in the impedance analysing software EC-Lab (Biologic, France), which is closely related to the anomalous open Warburg element proposed by Bisquert.^41^ The corresponding impedance expression reads5
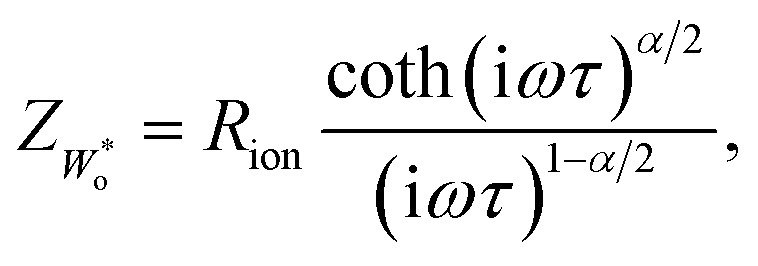
with a nonideality parameter 0 ≤ *α* ≤ 1. Thus, also in the present study, we replace the transmission line in Fig. 4b with the anomalous diffusion element 
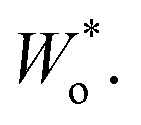
 The nonideality parameter was found to be in the range of 0.4 to 1. Furthermore, we replace the ideal double-layer capacitance *C*_dl_ by a constant-phase element *Q*_dl_ and add a serial high-frequency offset resistance *R*_hf_ to account for the ohmic resistance contributions of the electrolyte and other cell components.

A two-electrode cell setup is used in this study to avoid distortions of the impedance spectra that are potentially encountered in a three-electrode setup due to slight misalignments of the square-shaped working and counter electrodes. Hence, also the Li metal counter electrode must be included in the equivalent circuit. Its impedance is observed as a nearly invariant high-frequency semicircle, as shown in Fig. 3, and can therefore be accounted for by a *R*_CE_/*Q*_CE_ element in series to the remaining equivalent circuit with fixed values of *R*_CE_, *Q*_CE_, and the corresponding constant-phase exponent *n*. The side-reaction resistance *R*_s_ was also found to be nearly constant throughout the series of measurements and was therefore fixed to ensure a meaningful fit in the potential regions of high chemical capacitance, where the low-frequency diffusional tail is too short to allow an unambiguous identification of *R*_s_. The final equivalent circuit used for fitting is shown in Fig. 4c, with the fixed circuit elements marked in red. The corresponding fixed fit parameters for d-LNMO and o-LNMO, which were extracted from the impedance spectra at 3.90 V and 4.50 V, respectively, are summarised in Table 1. Exemplary impedance fits at 3.90 V are shown in Fig. 3d and h for d-LNMO and o-LNMO, respectively. A more extensive overview of the impedance fits is shown in Fig. S2 of the ESI.†

**Table 1:** Summary of fixed parameters for d-LNMO and o-LNMO impedance fits. The corresponding equivalent circuit elements are marked in red in Fig. 4c

	*R*_s_ [Ω]	*R*_CE_ [Ω]	*Q*_CE_ [F s^*n*−1^]	*n*
d-LNMO	1.95 × 10^5^	206	6.48 × 10^−6^	0.836
o-LNMO	2.45 × 10^5^	192	8.18 × 10^−6^	0.818

### Analysis of electrochemical properties

**Fig. 5:**
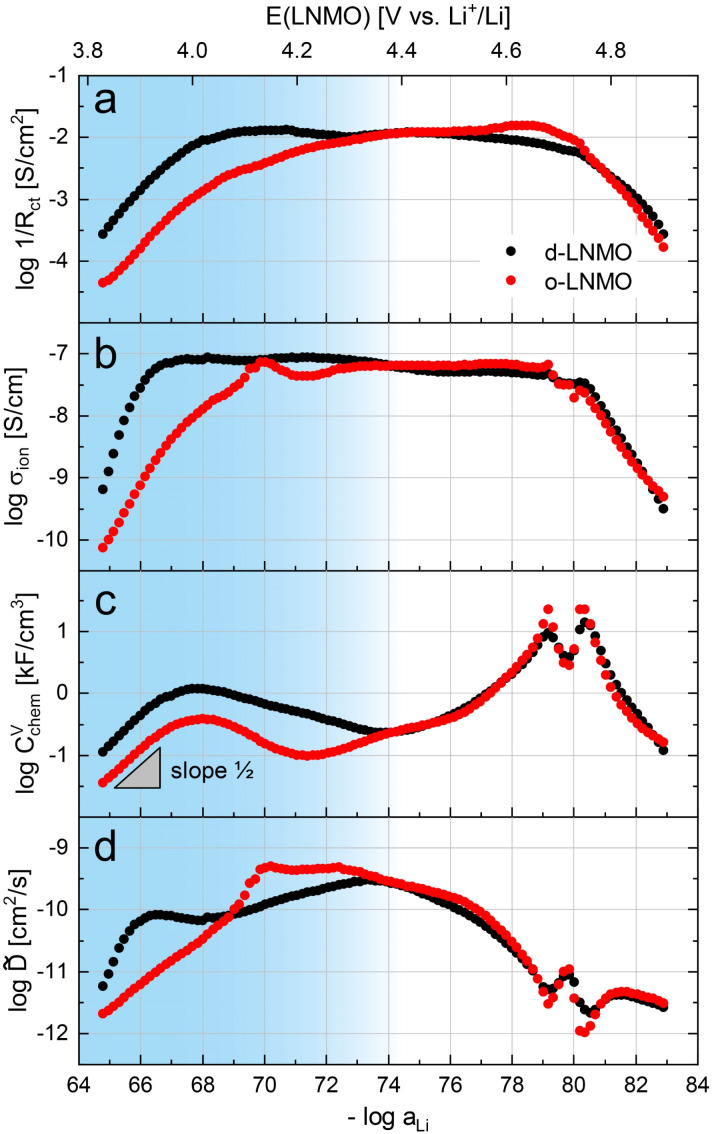
Logarithmic electrochemical properties of the d-LNMO (black) and o-LNMO (red) samples plotted as a function of negative logarithmic Li activity and electrode potential *E versus* Li^+^/Li. The voltage range coloured in blue corresponds to the oxygen vacancy regime, which is the main focus of this study, and where the most relevant differences between d-LNMO and o-LNMO are observed. (a) Inverse charge transfer resistance, (b) ionic conductivity, (c) volume-specific chemical capacitance, (d) chemical diffusion coefficient.

The electrochemical properties obtained by fitting the impedance spectra in Fig. 3 to the equivalent circuit from Fig. 4c are shown in Fig. 5 as a function of Li activity and electrode potential *versus* Li^+^/Li. The data sets of d-LNMO and o-LNMO are marked in red and black, respectively. For o-LNMO, impedance spectra at electrode potentials below 3.83 V *versus* Li^+^/Li could not be evaluated due to the high values of *R*_ct_ and *R*_ion_, which blend into each other in the low-frequency region and can no longer be distinguished by the fit (see, for example, the impedance spectrum at 3.80 V in Fig. 3e). The entire data set was therefore limited to an electrode potential range of 3.83 V to 4.90 V for both d-LNMO and o-LNMO.

The charge transfer resistance, normalised to the electrode surface area measured by AFM, is plotted as the inverse 1/*R*_ct_ in Fig. 5a to emphasise the similar concentration dependences of *σ*_ion_ and 1/*R*_ct_. For d-LNMO, *R*_ct_ decreases from 3.7 kΩ cm^2^ at 3.83 V to a minimum value of about 80 Ω cm^2^ at 4.14 V. From 4.05 V to 4.60 V, *R*_ct_ remains relatively constant around 100 Ω cm^2^. Above 4.60 V, *R*_ct_ starts to increase again and reaches a final value of 3.7 kΩ cm^2^ at 4.90 V. For o-LNMO, *R*_ct_ decreases from 23 kΩ cm^2^ at 3.83 V to about 100 Ω cm^2^ at around 4.33 V and then remains relatively constant up to 4.67 V, where it shows a slight further decrease down to a minimum of 70 Ω cm^2^, before increasing again up to 6.0 kΩ cm^2^ at 4.90 V. In the low potential region around 3.83 V to 4.15 V, *R*_ct_ is almost one order of magnitude higher for o-LNMO than for d-LNMO. From 4.15 V to 4.90 V, however, *R*_ct_ is very similar for o-LNMO and d-LNMO, with little variation between approximately 4.20 and 4.70 V. Overall, measured values of *R*_ct_ are in good agreement with literature.^42,43^

The SOC-dependent ionic conductivity *σ*_ion_ is plotted in Fig. 5b and shows a very similar variation with Li activity and electrode potential as *R*_ct_ for both d-LNMO and o-LNMO. In the range up to roughly 4.15 V, *σ*_ion_ is about one order of magnitude lower for o-LNMO than for d-LNMO. For d-LNMO, *σ*_ion_ increases by two orders of magnitude from 10^−9^ S cm^−1^ at 3.83 V to approximately 10^−7^ S cm^−1^ at 3.95 V. For o-LNMO, *σ*_ion_ even increases by three orders of magnitude from 10^−10^ S cm^−1^ to 10^−7^ S cm^−1^ at about 4.15 V. Above 4.15 V the ionic conductivity of both samples is nearly identical, remaining surprisingly constant up to 4.70 V and then decreasing again down to around 10^−9.5^ S cm^−1^ at 4.90 V. The broad plateau can be attributed to the presence of oxygen vacancies, as described in the defect chemical discussion. Literature reports of the SOC-dependent ionic conductivity are hard to find, but the value of about 10^−9^ S cm^−1^ measured by Amin and Belharouk for a nominally stoichiometric LNMO pellet^20^ is in good agreement with our results at the lowest electrode potentials (*i.e.*, close to full lithiation of the tetrahedral sites).

To provide a more direct comparison of d-LNMO and o-LNMO rate capabilities, the total effective electrode resistance *R*_tot_ = *R*_ion_/3 + *R*_ct_ is plotted in Fig. S3 of the ESI† as a function of electrode potential (Fig. S2a†) and Li content (Fig. S2b†). At low electrode potentials up to about 4.2 V *versus* Li^+^/Li, *R*_tot_ is about half an order of magnitude lower for d-LNMO than for o-LNMO, indicating a significantly better rate capability and energy efficiency for d-LNMO in this potential region. However, due to the small amounts of charge stored at low potentials, these differences are only significant close to full Li content (*i.e.*, the fully discharged state), as shown in Fig. S2b.†

The volume-specific chemical capacitance *C*^V^_chem_ of d-LNMO and o-LNMO is shown in Fig. 5c. Up to around 4.30 V *versus* Li^+^/Li, values of *C*^V^_chem_ are about half an order of magnitude higher for d-LNMO than for o-LNMO. For both samples, *C*^V^_chem_ exhibits a broad peak at 4.02 V, reaching a maximum value of 1185 F cm^−3^ and 382 F cm^−3^ for d-LNMO and o-LNMO, respectively. Above 4.30 V the data sets of both samples closely match each other, with the only difference being the sharper and higher double peaks of *C*^V^_chem_ for o-LNMO at 4.68 V and 4.74 V. The double peaks around 4.7 V strongly resemble the peaks found in LMO, presumably due to ordering of Li ions on the tetrahedral sites at half occupancy. A detailed analysis of these peaks is beyond the scope of this paper, which focusses on the broad peak around 4.0 V caused by oxygen vacancies. The general appearance and magnitude of our *C*^V^_chem_ measurements are in good agreement with CV curves and differential capacity plots found in the literature.^42–44^

**Fig. 6:**
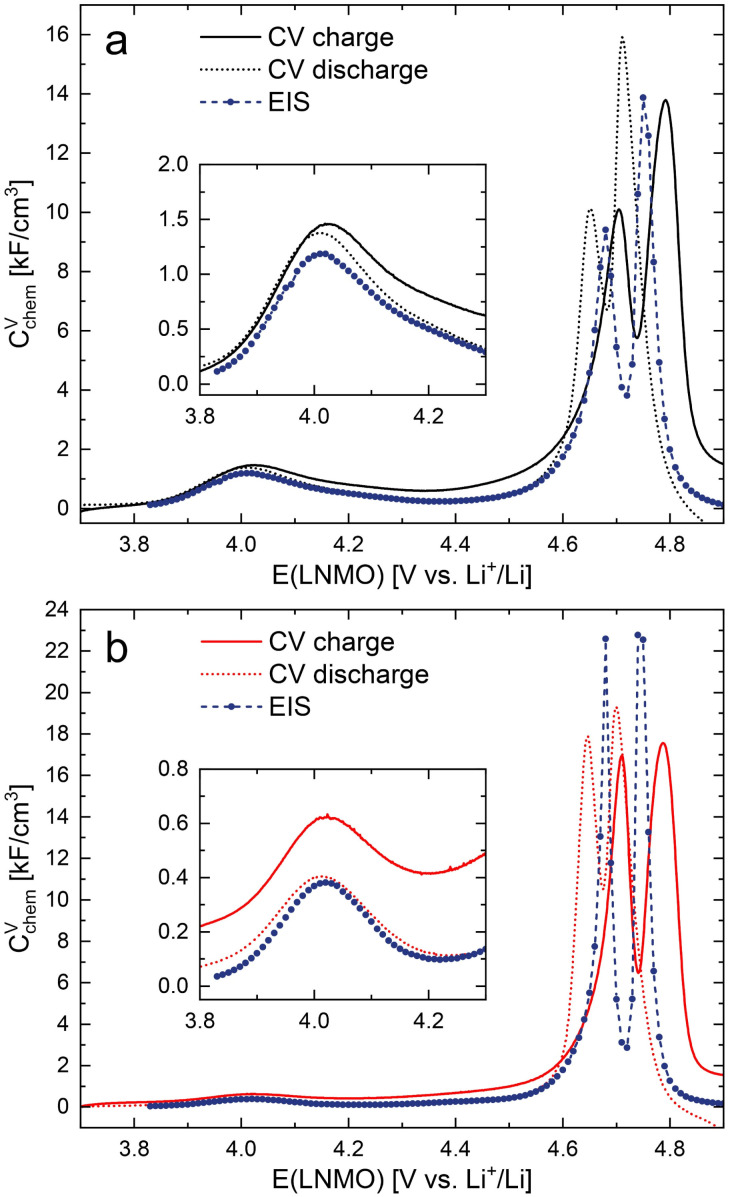
Comparison of the volume-specific chemical capacitance obtained from impedance fits (EIS) and from cyclic voltammetry (CV) scans for (a) d-LNMO and (b) o-LNMO.

The chemical capacitance from impedance fits can be compared to the values obtained from cyclic voltammetry *via*6
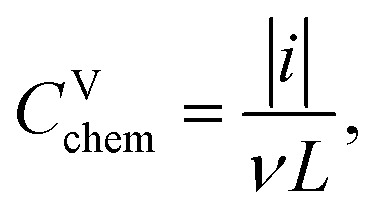
where *i* is the current density, *ν* the scan rate and *L* the film thickness. The *C*^V^_chem_ values from CV and impedance measurements are shown in a combined plot in Fig. 6a and b for d-LNMO and o-LNMO, respectively. Overall, the two data sets are in good agreement with each other for both samples, with the *C*^V^_chem_ values from CV scans being slightly higher than those from impedance measurements due to the presence of side reactions and corresponding background currents. Notably, for *C*^V^_chem_ from impedance data, the double peak around 4.7 V is confined to a smaller electrode potential range and appears in between the charge and discharge peaks of the CV scans. Both the broadening and shifting of the CV peaks with respect to the impedance data can be attributed to overpotentials encountered in the CV scans that increase with increasing current density and therefore are especially evident in regions of high chemical capacitance. These results again highlight the fact that the chemical capacitance of a Li storage thin film electrode, and hence also the equilibrium charge curve unaltered by kinetic overpotentials or side reactions, can be extracted from SOC-dependent impedance spectra.

The variation of the chemical diffusion coefficient *D̃* with Li activity and electrode potential (Fig. 5d) is more complex than the trends of *R*_ct_, *σ*_ion_, and *C*^V^_chem_, and needs to be understood as the ratio between *σ*_ion_ and *C*^V^_chem_ according to eqn (4). Without going into details of the differences between d-LNMO and o-LNMO, we can state that the *D̃* values are very similar above 4.4 V and significantly different for lower voltages. The latter is not surprising, considering the differences in *σ*_ion_ and *C*^V^_chem_ in the same potential region. The two sharp minima at 4.68 V and 4.74 V reflect the corresponding maxima of *C*^V^_chem_ at the same electrode potentials. As expected from the *C*^V^_chem_ curve, these minima are sharper and deeper for o-LNMO than for d-LNMO. The shape and magnitude of the variation of *D̃* with SOC agree excellently with other literature reports, although values below 4.3 V are rarely reported.^42,44,45^

Overall, the d-LNMO and o-LNMO samples behave similarly in the high-voltage region above approximately 4.30 V, where reversible Li^+^ release/insertion occurs at the tetrahedral Li sites upon oxidation/reduction of Ni^2+/3+/4+^. At potentials below 4.30 V, on the other hand, the strongly deviating electrochemical properties of the two samples clearly reflect the higher charge carrier concentrations of d-LNMO in the Mn^3+/4+^ regime due to a higher level of oxygen deficiency, which was already evident from the CV curves in Fig. 2a. We define the oxygen vacancy regime as the electrode potential region below 4.37 and 4.21 V for d-LNMO and o-LNMO, respectively, which corresponds to the position of the respective *C*^V^_chem_ minima in Fig. 6. By integrating the chemical capacitance according to7
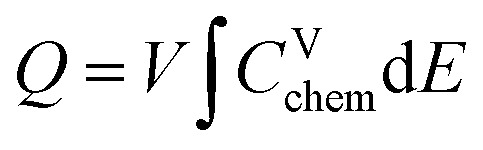
for the corresponding potential limits, the fraction of the total reversible capacity located in the Mn-regime can be evaluated as 19% and 4.2% for d-LNMO and o-LNMO, respectively. By reconsidering the explicit chemical formula LiNi_0.5_Mn^4+^_1.5−2*δ*_Mn^3+^_2*δ*_O_4−*δ*_, the corresponding oxygen nonstoichiometries can then be estimated as *δ* = 0.095 and *δ* = 0.021, which amounts to oxygen vacancy concentrations of 2.4% and 0.53% with respect to oxygen sites, respectively. Beside the differences in total capacity, the *C*^V^_chem_ curves of d-LNMO and o-LNMO in the oxygen vacancy regime also differ in their slope above 4.02 V, which will be analysed in more detail in the next section. As already discussed in the introduction, the cation/anion imbalance may also be due to cation interstitials, even though in literature mostly oxygen vacancies are assumed. The following defect chemical analysis of the electrochemical properties would then have to be adapted without changing its basic concepts and conclusions.

## Discussion

### Defect chemical model for oxygen-deficient LNMO

The material parameters deduced so far describe the kinetics of Li insertion (*R*_ct_, *σ*_ion_, *D̃*) and its thermodynamics (*C*^V^_chem_). For *σ*_ion_, and *C*^V^_chem_, a more or less straightforward relation to defect chemical properties and considerations can be expected. Particularly a defect chemical analysis of *C*^V^_chem_ may be a powerful tool for understanding and interpreting the impact of oxygen vacancies on the charge/discharge behaviour of LNMO. This is shown in the remaining part of the paper. First, we introduce the basic defect chemical model of stoichiometric and oxygen-deficient LNMO, which already qualitatively explains many features of the measured *C*^V^_chem_ (and *σ*_ion_) dependences on voltage. In a second step, we specify the defect model for the additional storage regime introduced by oxygen vacancies (oxygen vacancy regime). Finally, we compare the measured chemical capacitance with the model and refine the model such that all essential features (absolute value of *C*^V^_chem_ and slope shapes at both sides of the *C*^V^_chem_ peak) can be explained. This refined consideration leads to important conclusions with respect to the local chemical environment of oxygen vacancies and defect interactions between oxygen vacancies, lithium vacancies and electrons. Thus, it substantially improves the understanding of LNMO voltage profiles.

In a recent study on LMO thin films,^2^ it was shown that the defect chemistry of spinel cathode materials, *i.e.*, the ionic and electronic point defect concentrations and chemical capacitance as a function of electrode potential, can be described by dilute-solution thermodynamics over a surprisingly wide Li stoichiometry range when taking site restriction effects into account. By solving the set of mass action laws defined by the available octahedral and tetrahedral lattice sites, together with the appropriate charge neutrality expression, the corresponding point defect concentrations as a function of Li activity (*i.e.*, a Brouwer diagram) can be obtained. As long as only one type of Li site and one type of redox couple need to be considered, an analytical expression for *C*^V^_chem_ can be derived, which depends only on the relevant defect concentrations. As soon as two or more lattice sites become relevant, such as the octahedral and tetrahedral sites in a typical spinel material, *C*^V^_chem_ can only be calculated *via*eqn (1) and therefore requires detailed knowledge of the Li chemical potential *μ*_Li_ and its defect concentration dependences. For oxygen-deficient LNMO, this defect chemical description is further complicated by (i) the presence of a second type of transition metal redox couple Ni^2+/3+/4+^ in addition to Mn^3+/4+^ (ii) the presence of oxygen vacancies that act as donor dopants and (iii) the potential of these oxygen vacancies to engage in charge trapping (*i.e.*, defect association) reactions. The general formalism for calculating (i) the total Li chemical potential, and thus also *C*^V^_chem_, for multiple Li sites, multiple redox couples and variable doping states, as well as (ii) the relevant defect concentrations as a function of electrode potential is detailed in Section 4 of the ESI.† Herein, we restrict ourselves to a qualitative discussion of the resulting Brouwer diagrams under certain assumptions.

**Fig. 7:**
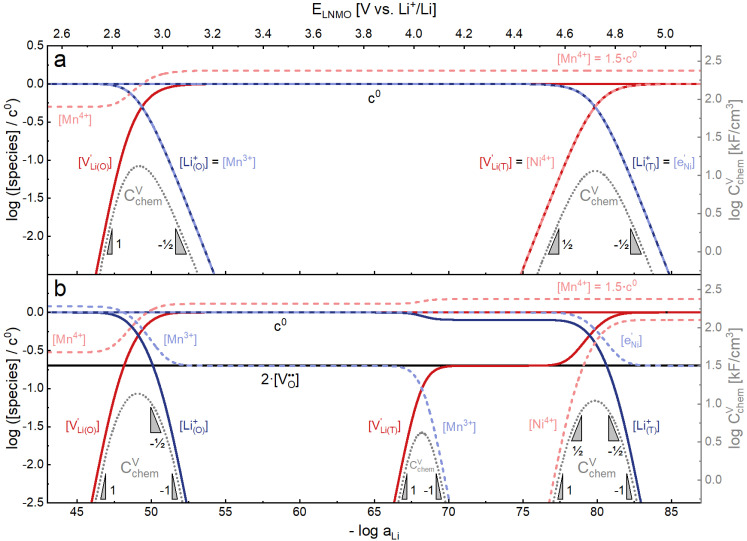
Calculated Brouwer diagrams of (a) stoichiometric and (b) oxygen-deficient LNMO, neglecting Li ordering on tetrahedral sites and thus the characteristic double peak at 4.7 V. For both diagrams, charge carrier concentrations were calculated *via* eqn (S6), (S7), (S10), and (S11) in the ESI.†*C*^V^_chem_ was calculated from eqn (1) by inserting *μ*_Li_ as derived in the ESI (eqn (S3)–(S9)).† The values 
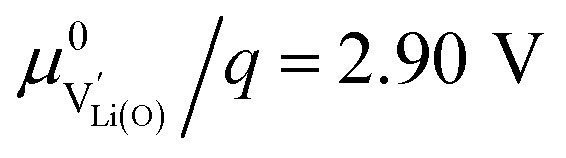
, 
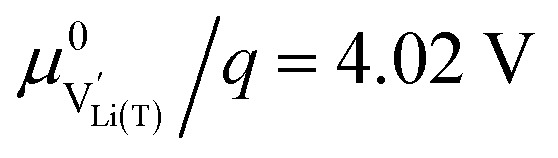
, 
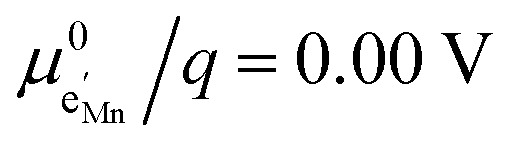
, and 
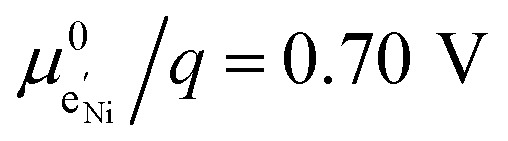
 were chosen such that the chemical capacitance peaks of the defect model occur around the same electrode potentials as observed experimentally. For the defect model of oxygen-deficient LNMO, an oxygen deficiency of *δ* = 0.1 was inserted into eqn (S10).† Please note that the continuous octahedral regime around 2.9 V is not observed experimentally due to the presence of a two-phase region.

To simplify our brief discussion of the Ni regime around 4.7 V, we consider neither separate Ni^2+/3+^ and Ni^3+/4+^ redox regimes nor Li ordering. We treat Ni as a single type of redox couple contributing to the total capacity with one electron per formula unit, instead of considering it as two different redox couples, each contributing 0.5 electrons per formula unit. In the following analysis, an electron 
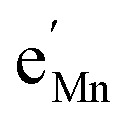
 on Mn corresponds to Mn^3+^ and an electron 
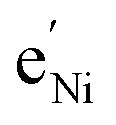
 on Ni corresponds to 
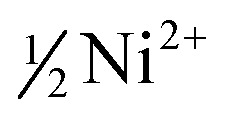
, with 
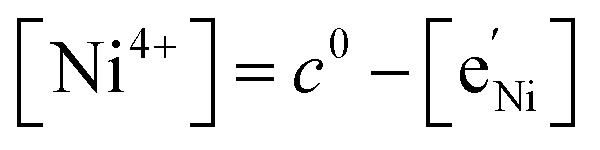
 and *c*^0^ being the concentration of formula units. In a first step, we discuss the defect concentrations as a function of Li activity (*i.e.*, the Brouwer diagram), assuming a single tetrahedral site regime without ordering effects. For the sake of illustration, we also include a continuous octahedral site regime without any two-phase regions, even though this is not observed in experiments. The resulting Brouwer diagram is shown in Fig. 7a.

As also shown for LMO,^2^ the Brouwer diagram of stoichiometric LNMO features two main storage regimes that correspond to the octahedral and tetrahedral sites. However, in LNMO there are two different transition metals (Mn and Ni) with redox capacities of 1.5 and 1 electron per formula unit, respectively, rather than a single transition metal (Mn) that covers the total storage capacity of two formula units in LMO. As a consequence, the voltage-dependent concentrations of electrons show a similar site-restricted behaviour as the ionic charge carriers, that is, Li vacancies and Li^+^ on the Li sites. In the tetrahedral site regime without ordering (around 4.7 V), the Brouwer diagram of stoichiometric LNMO resembles that of a generic layered oxide (see ref. 2, Fig. 7a). At high and low tetrahedral site occupancy, 
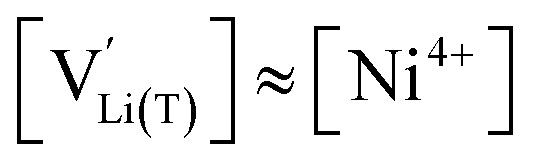
 and 
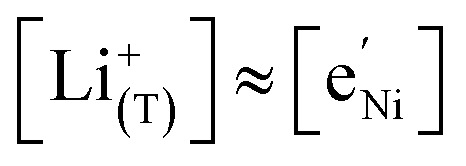
 vary with slopes of ½ and −½, respectively, with a corresponding *C*^V^_chem_ peak in the transition region around half site occupancy, where 
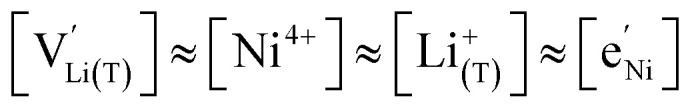
. Hence, the chemical capacitance peak originates from the site restriction of both Li vacancies on the tetrahedral sites and electrons on Ni. In the hypothetical octahedral site regime around 2.9 V, 
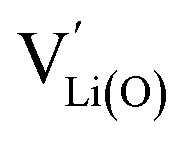
 increases with a slope of 1 at high site occupancy with [Mn^4+^] ≈ 0.5*c*^0^, while [Li^+^_(O)_] ≈ [Mn^3+^] varies with a slope of −½ at low site occupancy. These slopes are again reflected in the corresponding *C*^V^_chem_ peak. It is also straightforward to include Li ordering in the tetrahedral regime by considering two energetically different tetrahedral sites. This was extensively discussed for pure LMO and can explain the experimentally observed characteristic double peak around 4.7 V.^2^ In this study, however, we focus on the effects introduced by oxygen vacancies, *i.e.*, donor doping.

Fig. 7b displays the Brouwer diagram for such an oxygen-deficient material, again without Li ordering on the tetrahedral sites. Assuming that oxygen vacancies are electronically compensated by Mn^3+^ according to 
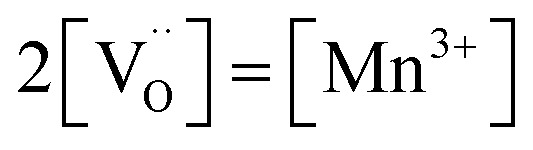
 for LiNi_0.5_Mn^4+^_1.5−2*δ*_Mn^3+^_2*δ*_O_4−*δ*_, the tetrahedral site regime is split into a low-voltage Mn (4.0 V) and a high-voltage Ni (4.7 V) subregime, as shown in Fig. 7b. Interestingly, the *C*^V^_chem_ peaks now are no longer caused solely by Li and/or electron site restriction, but reflect transitions from ionically to electronically dominated charge compensation of the oxygen vacancies. More specifically, in the oxygen vacancy regime around 4.0 V, charge compensation switches from [Mn^3+^] to 
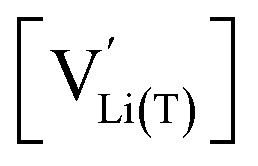
, with slopes of 1 and −1, respectively, and a corresponding peak in *C*^V^_chem_. The total storage capacity of this regime is defined by the oxygen vacancy concentration. Please note that this situation is fully analogous to the chemical capacitance peak observed in acceptor-doped mixed conducting oxides, where charge compensation switches from oxygen vacancies to electron holes when increasing the oxygen chemical potential.^46^

In the tetrahedral-site Ni regime around 4.7 V, the oxygen vacancies remain compensated by Li vacancies, resulting in slopes of 1 and −1 for [Ni^4+^] and [Li^+^_(T)_], being the minority charge carriers at high and low site occupancy, respectively. At intermediate site occupancies, where 
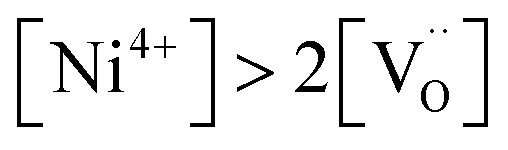
 and 
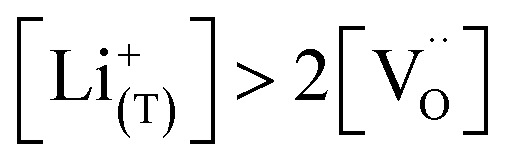
, the slopes flatten from 1 and −1 to ½ and −½ for 
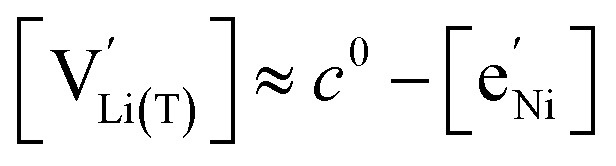
 and 
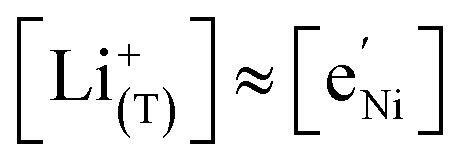
, respectively. For the entire hypothetical octahedral site regime, oxygen vacancies remain compensated by [Mn^3+^], resulting in a steepening of the slope of [Li^+^_(O)_] from −½ to −1 for 
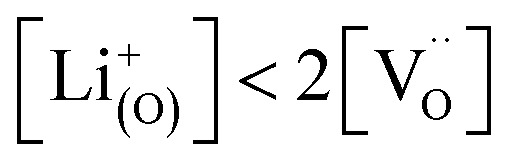
. Since a total of 1.5 Mn^4+^ are available per formula unit compared to only 1 octahedral Li site, the total capacity of the octahedral regime is not affected for realistic levels of oxygen deficiency.

Qualitatively, the tetrahedral regime (approximately 3.7 V to 5.0 V) of the Brouwer diagram for oxygen-deficient LNMO in Fig. 7b can explain several key features of the experimental data presented in Fig. 5. The inverse charge transfer resistance and the ionic conductivity both increase and decrease at low and high electrode potentials, respectively, but remain nearly constant at intermediate potentials from about 4.1 V to 4.6 V. This constancy of 1/*R*_ct_ and *σ*_ion_ is remarkably consistent with the almost constant concentration of the relevant ionic charge carrier 
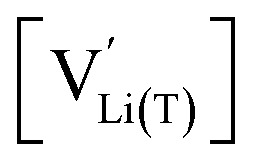
 between the 4.0 V and 4.7 V regimes. Both 1/*R*_ct_ and *σ*_ion_ are lower for o-LNMO than for d-LNMO in the low-voltage region due to the lower oxygen vacancy and hence lower Li vacancy concentrations in this potential region. The fact that this difference almost vanishes at intermediate electrode potentials suggests a difference in the concentration-dependence of the respective ionic mobilities of d-LNMO and o-LNMO.

As predicted by Fig. 7b, *C*^V^_chem_ exhibits two main peaks corresponding to the tetrahedral-site Mn and Ni regimes around 4.0 V and 4.7 V, respectively, the former being lower for o-LNMO due to the lower oxygen vacancy concentration. As already discussed above, the pronounced double peak measured at 4.68/4.74 V is attributed to Li ordering on tetrahedral sites, as for pure LMO.^2^ This effect is not included in the calculations of Fig. 7b and beyond the scope of this paper. However, at the onset of the Ni regime, around 4.6 V, both samples show a *C*^V^_chem_ slope of ½, in agreement with Fig. 7b for high charge carrier concentrations. The region of low [Ni^4+^], where a slope of 1 would be expected, is not visible in the experimental data due to an overlap with the smeared out low-voltage oxygen vacancy regime (see below). In the following, we analyse the impact of oxygen deficiency on the defect chemistry of LNMO in more detail and therefore focus on the oxygen vacancy regime (Mn regime) below 4.5 V.

### Defect chemical model for the oxygen vacancy regime

We start by isolating the oxygen vacancy regime (*i.e.*, the tetrahedral-site Mn regime introduced through charge compensation of oxygen vacancies) from the full defect chemical model in Fig. 7b. Since only one Li site (tetrahedral site) and one redox couple (Mn^3+/4+^) are relevant in this regime, an explicit expression for the Li chemical potential can easily be derived, without resorting to the more complicated multi-site formalism in Section 4 of the ESI.† For this purpose, we consider the Li insertion equilibrium8
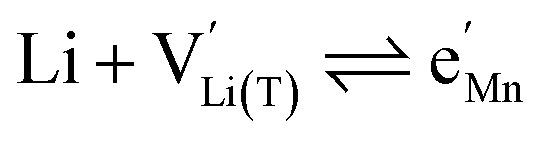
for the relevant defects, that is, tetrahedral Li vacancies and electrons, in Kröger–Vink notation, with 
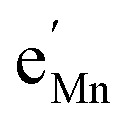
 corresponding to Mn^3+^. To account for different capacities, and thus also different site restrictions, of lattice sites and redox species, we write the site occupancy *x*_*j*_ of species *j* as9
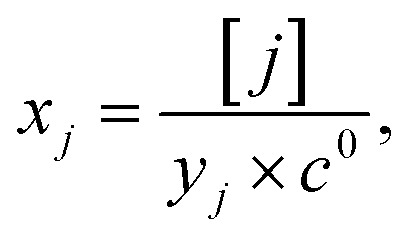
with [*j*] and *y*_*j*_ being the concentration of species *j* and the number of corresponding sites per formula unit, respectively. For example, 
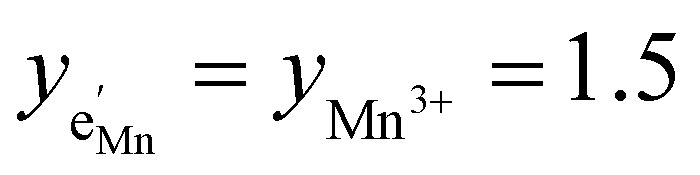
 and *y*_Ni^4+^_ = 0.5. The balance of chemical potentials can then be written as10
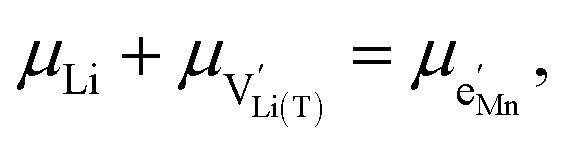
where 
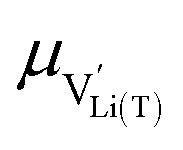
 and 
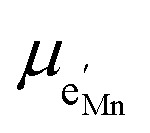
 are the individual site-restricted chemical potentials of tetrahedral vacancies and electrons (or Mn^3+^), which are related to the respective site occupancies according to11
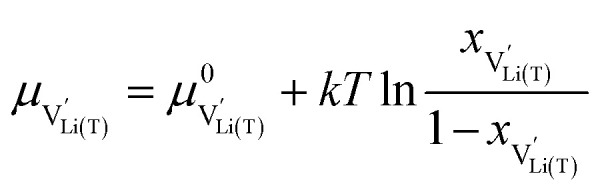
and12
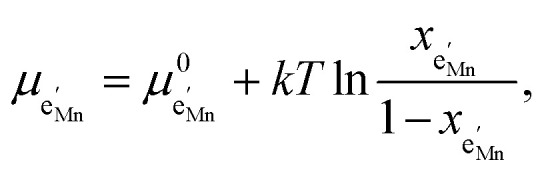
with *μ*^0^_*j*_ being the standard chemical potential of species *j*. By combining eqn (2) and (10)–(12), the corresponding law of mass action can then be formulated as13
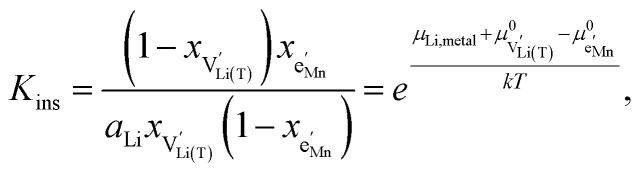
with *K*_ins_ being the equilibrium constant of Li insertion according to eqn (10). This shows that *K*_ins_ only depends on the standard chemical potentials of tetrahedral Li vacancies and electrons relative to Li metal. In the case of oxygen-deficient LNMO, the charge neutrality expression reads14
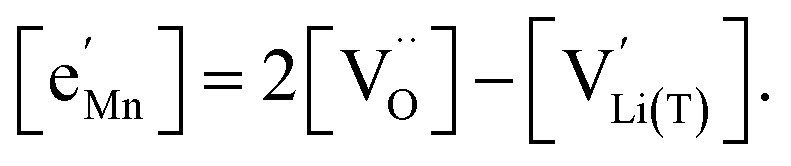
The concentrations of Mn^3+^, Mn^4+^, 
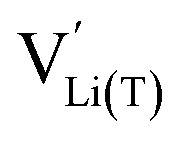
 and Li^+^_(T)_ are related *via*15
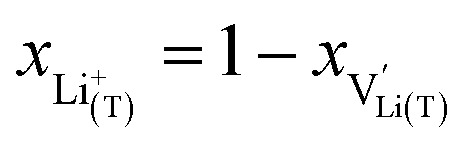
and16
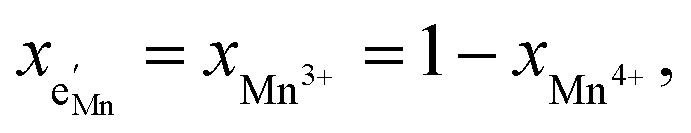
with 
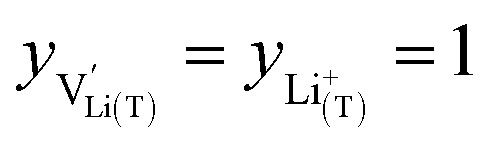
 and *y*_Mn^3+^_ = *y*_Mn^4+^_ = 1.5. The system of equations defined by eqn (13)–(16) can then be solved for the four individual point defect concentrations as a function of Li activity if *K*_ins_ or the corresponding standard chemical potentials *μ*^0^_*j*_ are known.

Furthermore, the chemical capacitance can be obtained by inserting eqn (9)–(12) and (14) into eqn (1). For realistic oxygen vacancy concentrations, *i.e.*, for *δ* ≪ 1, site restriction of 
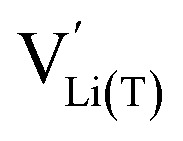
 and 
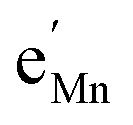
 can be neglected and eqn (11) and (12) can be simplified by assuming 
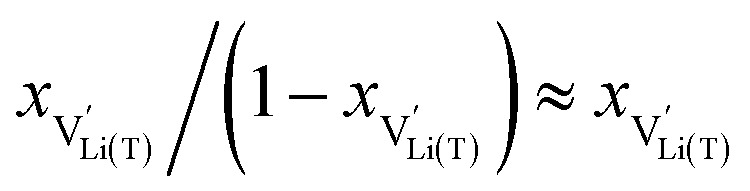
 and 
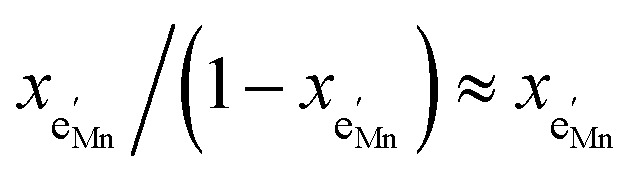
. In this case, the chemical capacitance can be expressed as^37^17
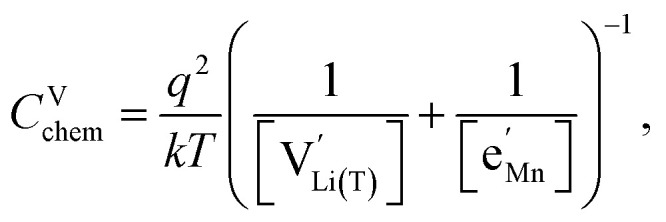
which reflects the behaviour of the chemical capacitance as a serial double capacitor, with an effective capacitance determined by the smaller of the concentrations of tetrahedral Li vacancies 
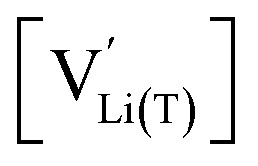
 and Mn^3+^
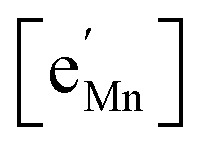
.

**Fig. 8:**
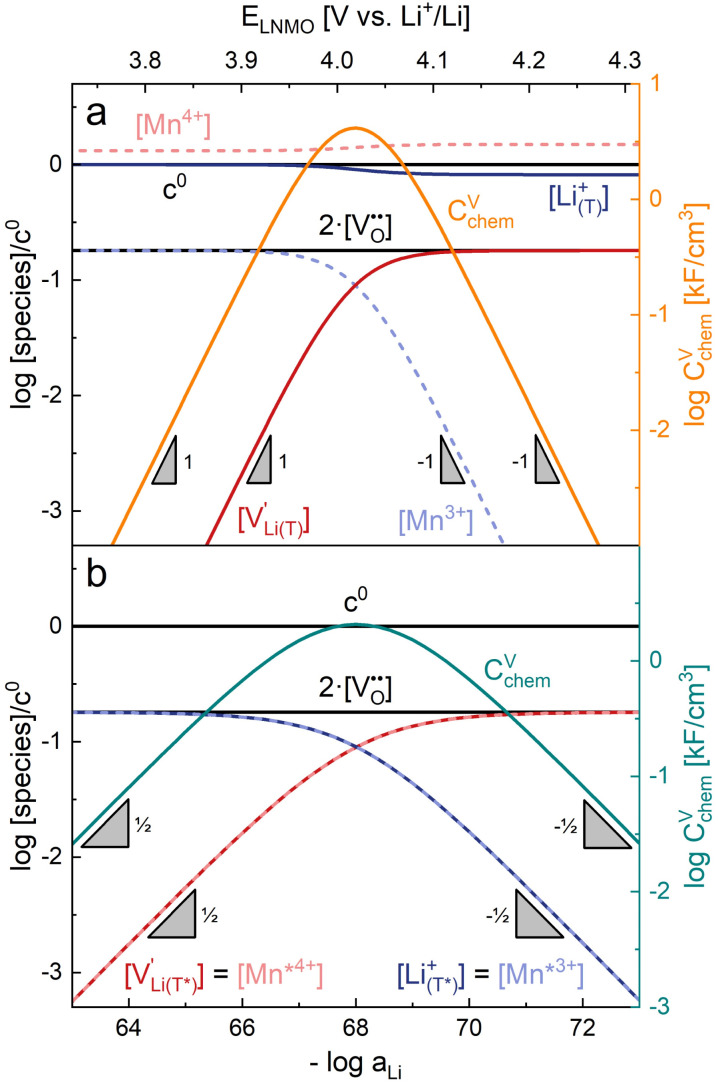
(a) Brouwer diagram and chemical capacitance of the oxygen vacancy regime extracted from Fig. 7b for 
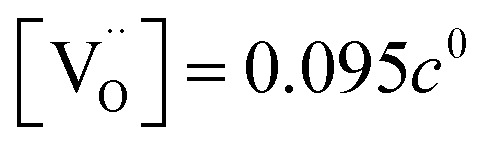
 and *K*_ins_ = 10^−67.8^. (b) Brouwer diagram and chemical capacitance of the oxygen vacancy regime, assuming energetically non-equivalent Li_T*_ and Mn* sites, for 
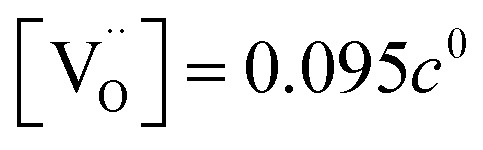
 and *K*_ins_ = 10^−68^.

The resulting point defect concentrations and chemical capacitance are shown in a log–log plot *versus* −log *a*_Li_ (*i.e.*, a Brouwer diagram) in Fig. 8a for 
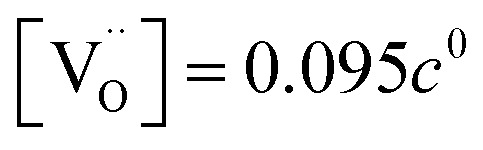
 and *K*_ins_ = 10^−67.8^. The value of 
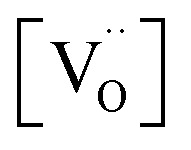
 is taken from the integration of the d-LNMO *C*^V^_chem_ data of impedance fits below 4.37 V (see eqn (7) and following paragraph). *K*_ins_ is chosen such that the *C*^V^_chem_ peak of the defect model appears at the same electrode potential *versus* Li^+^/Li as in the experimental results. Due to the clear separation of the octahedral (2.9 V), oxygen vacancy (4.0 V) and Ni tetrahedral (4.7 V) regimes in Fig. 7b, the isolated defect model of the oxygen vacancy regime (Fig. 8a) is virtually identical to the full defect model (Fig. 7b) of oxygen-deficient LNMO in the selected potential region. The Brouwer slopes of 1 and −1 in the chemical capacitance result from the corresponding variation of 
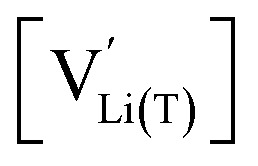
 and [Mn^3+^] 
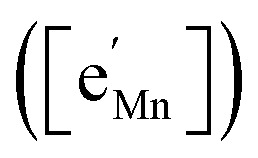
, respectively, while [Li^+^_(T)_] and [Mn^4+^] remain nearly constant on a logarithmic scale.

**Fig. 9:**
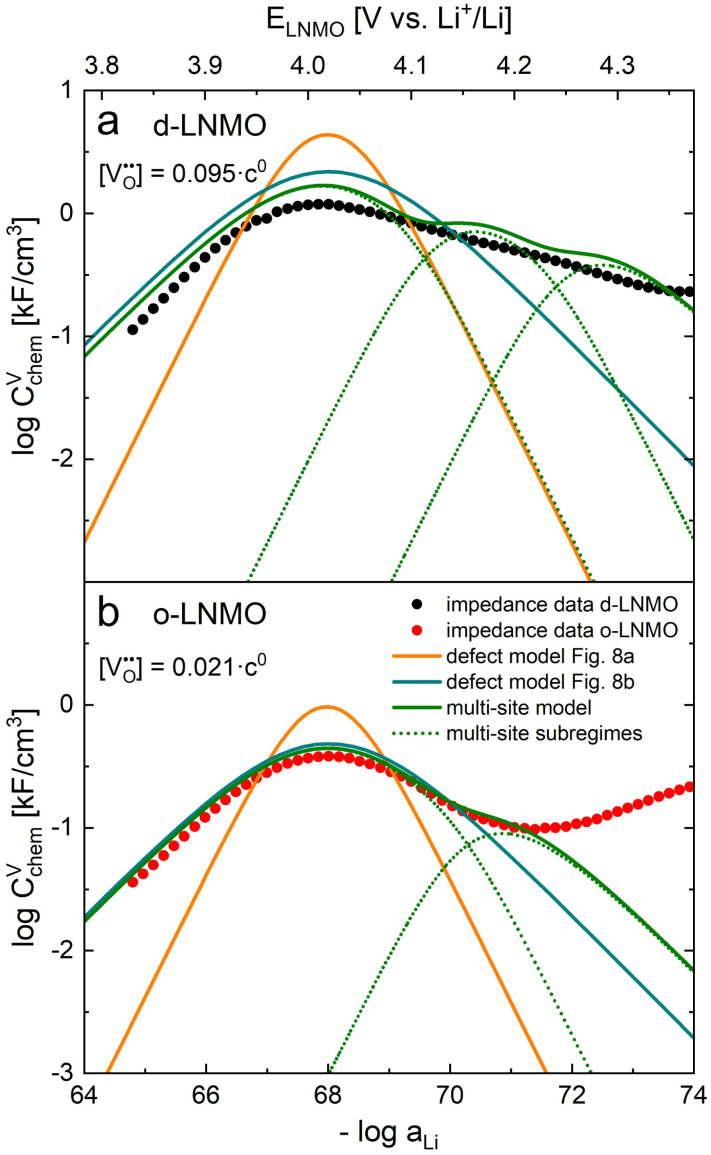
Calculated chemical capacitance of three different defect models (continuous lines) compared to the data from impedance fits (black dots). (a) d-LNMO: experimental data and calculated chemical capacitances from Fig. 8a, b and from the multi-site model for 
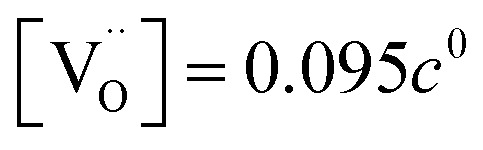
, *p*_1_ = 0.66, *p*_2_ = 0.21, *p*_3_ = 0.13, 
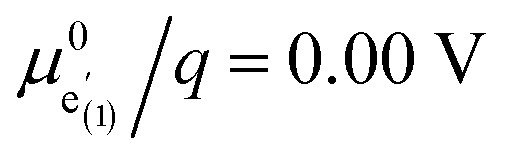
, 
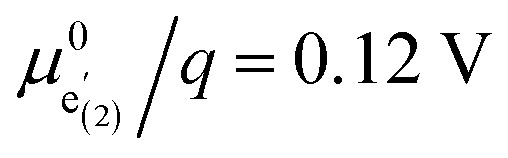
, 
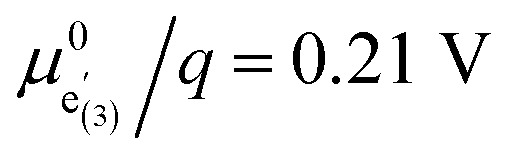
, and 
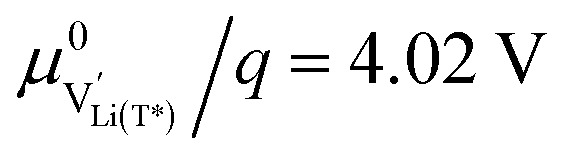
. (b) o-LNMO: experimental data and calculated chemical capacitances from Fig. 8a, b and from the multi-site model for 
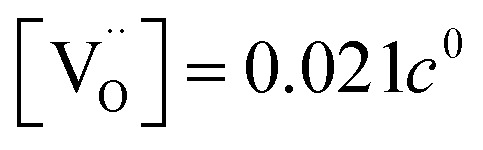
, *p*_1_ = 0.86, *p*_2_ = 0.14, 
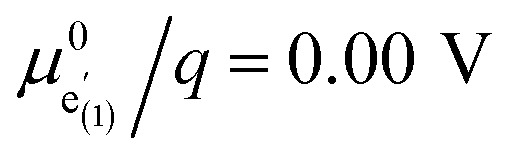
, 
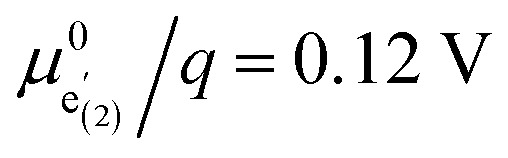
, and 
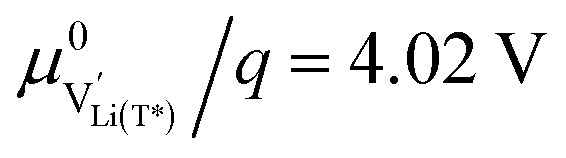
.

A direct comparison of the chemical capacitance from impedance fits (black dots) and from the defect model in Fig. 8a (orange line) is shown in Fig. 9a and b for d-LNMO 
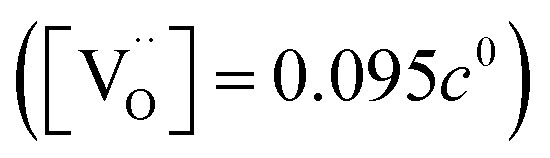
 and o-LNMO 
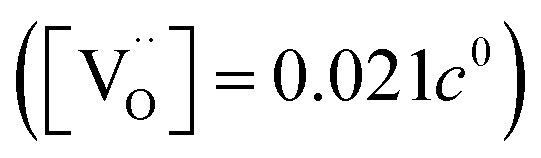
, respectively. For both o-LNMO and d-LNMO, the defect model from Fig. 8a predicts a *C*^V^_chem_ peak, the position of which reflects the value of *K*_ins_. The total charge contained within the *C*^V^_chem_ peak corresponds to 
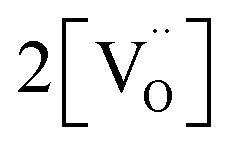
, and enters the defect chemical calculation as a fixed parameter derived *via* integration of the experimental *C*^V^_chem_ curve up to the minimum between the oxygen vacancy and Ni regimes *via*eqn (7). Although the calculated *C*^V^_chem_ curve appears to be in the same order of magnitude as the experimental data in terms of total charge (*i.e.*, area under the peak), the calculated curve exhibits steeper slopes and significantly higher peak values for both d-LNMO and o-LNMO. Furthermore, the model fails to describe the particularly flat slope of the experimental data between 4.0 and 4.3 V. In the following, we refine the defect model by including different Li site energies close to oxygen vacancies.

### Chemical capacitance indicating modified tetrahedral Li sites in the oxygen vacancy regime

The defect model in Fig. 8a assumes that the tetrahedral Li sites and Mn^3+/4+^ redox centres being active in the oxygen vacancy regime around 4.0 V are indistinguishable from the tetrahedral Li sites involved in the tetrahedral-site Ni regime around 4.7 V and the remaining Mn sites, respectively. In other words, it is assumed that the local chemical environments surrounding each point defect are unaffected by the oxygen vacancies. However, owing to electrostatic considerations, it is reasonable to assume that Li vacancies and Mn^3+^ are significantly stabilized in vicinity to an oxygen vacancy. This leads us to conclude that a suitable defect chemical model for d-LNMO has to consider not only the presence of Mn^3+^ due to charge compensation of the oxygen vacancies, but also the different local chemical environments of the ionic and electronic point defects involved in the corresponding oxygen vacancy regime.

As a first step to include defect interactions, we adapt our defect model to treat the tetrahedral Li sites and Mn^3+/4+^ species involved in the oxygen vacancy regime as energetically non-equivalent to the remaining Li sites and Mn redox centres. Accordingly, we assume that Li storage in the oxygen vacancy regime is locally restricted to the lattice sites in immediate proximity to the oxygen vacancies. In a first approximation, this can simply be done by relating the number of available tetrahedral lattice sites T* or electronic sites Mn* per formula unit to the oxygen nonstoichiometry *δ* according to *y*_T*_ = *y*_Mn*_ = 2*δ*, where the asterisk denotes the non-equivalence of 
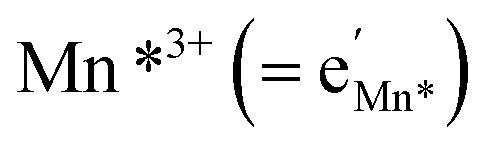
, Mn^4+^, 
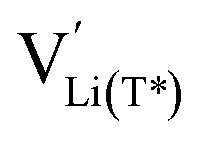
, Li^+^_(T*)_ and the corresponding defect species in Fig. 8a. Eqn (8)–(16) are then still valid for species j*, and can again be solved for the individual point defect concentrations [j*], with the additional condition18
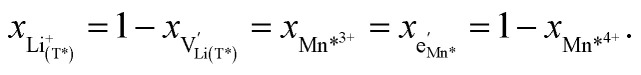
However, then site restriction can no longer be neglected, meaning that the full site-restricted chemical potentials in eqn (11) and (12) have to be inserted into eqn (1), together with the balance of chemical potentials (eqn (10)) to obtain the correct expression for *C*^V^_chem_, which reads19
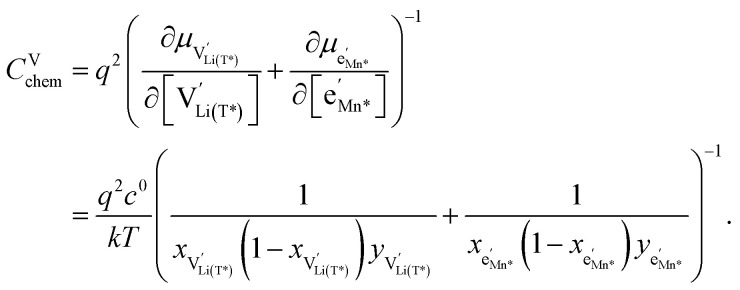


For 
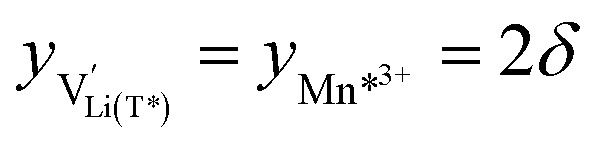
, eqn (19) simplifies into20
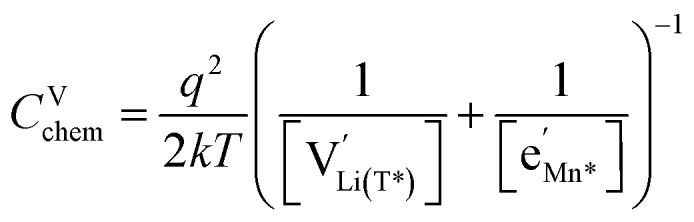
in analogy to eqn (17). Please note that in this case, a factor 2 results in the denominator of eqn (20). The resulting point defect concentrations are plotted together with *C*^V^_chem_ from eqn (20) as a Brouwer diagram in Fig. 8b for 
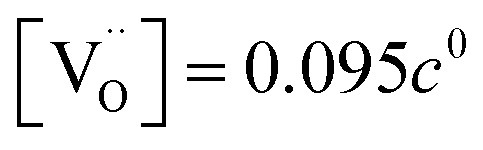
 and *K*_ins_ = 10^−68^. While in the original defect model in Fig. 8a, only 
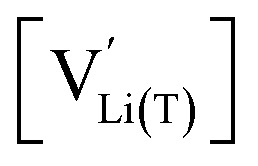
 and [Mn^3+^] vary significantly and cause a relatively sharp *C*^V^_chem_ peak with slopes of 1 and −1, the concentrations 
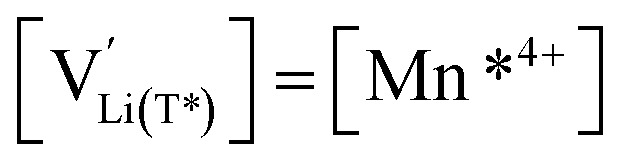
 and 
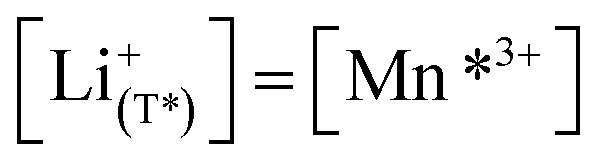
 vary in concert in the adapted defect model in Fig. 8b, resulting in a relatively broad *C*^V^_chem_ peak with slopes of ½ and −½. The total storage capacity, *i.e.*, the total area under the *C*^V^_chem_ curves, is the same for both models and is defined by the oxygen vacancy concentration in the material. Please note that due to the asymmetry of the defect model in Fig. 8a, the peak of *C*^V^_chem_ appears at −log *a*_Li_ > −log *K*_ins_, while in the adapted (now symmetric) defect model the *C*^V^_chem_ peak position is defined by −log *a*_Li_ = −log *K*_ins_. We therefore chose a slightly lower *K*_ins_ value for the defect model in Fig. 8a so that for both models the *C*^V^_chem_ peak is located at approximately −log *a*_Li_ = 68 (*E*_LNMO_ ≈ 4.02 V *versus* Li^+^/Li), where it is observed experimentally.

The comparison of the two different defect models with the experimental *C*^V^_chem_ data is shown in Fig. 9a and b for d-LNMO and o-LNMO, respectively. The adapted defect model clearly fits the experimental data better, in terms of both *C*^V^_chem_ slopes and absolute values. In the low-potential region below 4.0 V *versus* Li^+^/Li, the adapted model comes remarkably close to the experimental data, although the absolute values predicted by the model are slightly higher.

Interestingly, the Mn(*)^3+/4+^ transition and thus the *C*^V^_chem_ peak of the oxygen vacancy regime in LNMO is very close, in terms of electrode potential, to the main Mn^3+/4+^ transition in LMO. This requires very similar *K*_ins_ values. However, we already discussed that significant stabilisation of Li vacancies and electrons in the vicinity of oxygen vacancies are required to explain our measured *C*^V^_chem_ data. According to eqn (13), a similar equilibrium constant *K*_ins_ of LNMO and LMO despite very different standard chemical potentials 
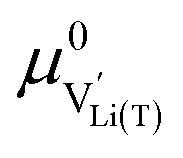
 and 
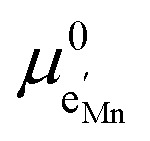
 is only possible if changes of the two standard chemical potentials are very similar.

However, in the high-potential region above 4.0 V, there are still substantial deviations of the model from the measured *C*^V^_chem_ values, with the calculated peak value being significantly higher and the slope being steeper than the experimental data. Especially for d-LNMO, the measured *C*^V^_chem_ peak appears smeared-out towards high potentials, with a slope that is significantly flatter than predicted by both defect models in Fig. 8. It is worth mentioning that this smeared-out *C*^V^_chem_ peak of d-LNMO is also seen in various other studies in the literature,^11,13,47–49^ but has received relatively little attention so far. In the following, we explain these deviations by leaving the assumption of only one 
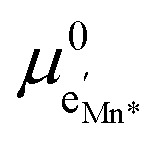
 level.

### Chemical capacitance as a fingerprint of stabilised Mn^3+^

So far, our defect model explains the absolute values of the *C*^V^_chem_ peak as well as its flattened slope close to ½ for voltages below 4.0 V. We still have to understand the even smaller slope above 4.0 V and the very broad decrease for the d-LNMO sample with a high concentration of oxygen vacancies.

In principle, we face a complex situation with multiple-defect interaction including (at least) oxygen vacancies 
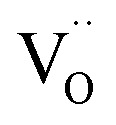
, lithium vacancies 
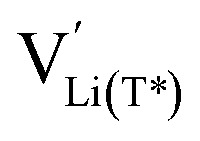
 or occupied Li sites Li^+^_(T*)_, and electrons Mn*^3+^. Different concepts may be employed for dealing with defect interactions, for example, activity coefficients or defect association equilibria. Owing to the complex situation with three relevant particles/defects, we choose a third approach to extend the given model with modified site energies around an oxygen vacancy. We simply introduce a finite number of different standard defect energies and thus different *μ*^0^ terms.

In this manner, we consider the existence of multiple energetically different lattice sites in proximity to oxygen vacancies, where Li storage is assumed to be located for the oxygen vacancy regime. The variations in the local chemical environments of these sites can modulate the stabilizing effect of the oxygen vacancy on both Li vacancies and electrons (Mn*^3+^). Additional stabilization of Li vacancies would move part of the chemical capacitance to lower electrode potentials, as the corresponding Li^+^_(T*)_ is more easily removed from the lattice. For electrons, on the other hand, additional stabilization would shift part of the chemical capacitance to higher electrode potentials, as the stabilized Mn*^3+^ is less easily oxidised. Since the defect model in Fig. 8b is already in good agreement with the measured data below 4 V, but still shows significant deviations above 4 V, especially for d-LNMO (*c.f.*Fig. 9a), we refine the defect model only for electrons to account for different local chemical environments in vicinity to an oxygen vacancy.

For this purpose, we resort to the multi-site formalism described in Section 4 of the ESI.† More specifically, we consider the presence of three (d-LNMO) or two (o-LNMO) types of Mn* sites, each 
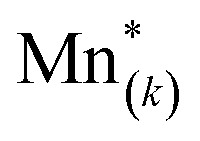
 site having a different value of 
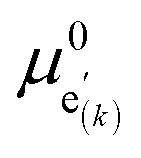
, and derive the corresponding multi-site-restricted electron chemical potential *μ*_e′_ from eqn (S7) and (S9).† Since the total number of available Li_(T*)_ and Mn* sites is the same and no additional doping effects need to be considered within the oxygen vacancy regime itself, the correction term *c* in eqn (S9)† can be set to zero. To assign each of the Mn* sites a fraction of the total oxygen vacancy regime capacity 2*δ*, the number of available lattice sites per formula unit can be defined as 
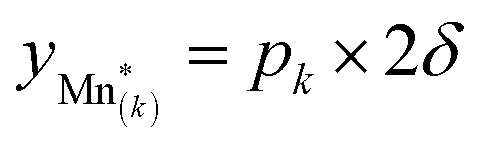
, with 0 ≤ *p*_*k*_ ≤ 1, ∑*p*_*k*_ = 1, and 
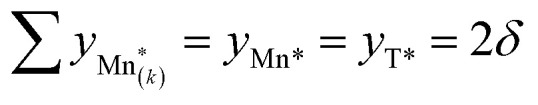
. Thus, the *p*_*k*_ factors indicate the fraction of the total oxygen vacancy regime capacity that is attributed to each 
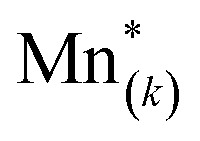
 site. The resulting *μ*_e'_ can then be inserted into eqn (10), together with the unchanged 
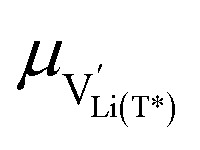
 from eqn (11), to obtain the total Li chemical potential *μ*_Li_ and finally the chemical capacitance *via*eqn (1).

The resulting chemical capacitance curves (green line) are shown in comparison to the experimental data and the previous defect models in Fig. 9a and b for d-LNMO (*δ* = 0.095) and o-LNMO (*δ* = 0.021), respectively. The corresponding subregimes for each 
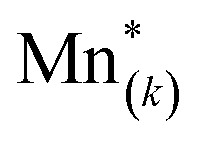
 site are indicated as dotted lines. For both plots, the values of 
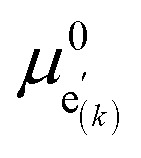
 and *p*_*k*_ were chosen manually to approximate the experimental data, as indicated in the figure caption. Please note that, although a mathematical fitting algorithm could in principle be applied to obtain the closest approximation, the conclusions of our qualitative discussion would remain the same. For both d-LNMO and o-LNMO, the calculated *C*^V^_chem_ curves are in good agreement with the experimental data for the entire relevant voltage range (up to 4.37 and 4.21 V *versus* Li^+^/Li for d-LNMO and o-LNMO, respectively).

As expected for a multi-site model, the *C*^V^_chem_ peaks are now split into three/two sub-peaks for d-/o-LNMO. For both samples, the model features sub-peaks at 4.02 V and 4.17 V, with d-LNMO having an additional sub-peak at 4.28 V. Each sub-peak corresponds to a different 
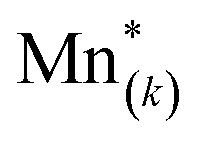
, with the total capacity remaining the same as for defect models from Fig. 8a and b. The relative capacities of the peaks were set to *p*_1_ = 0.66, *p*_2_ = 0.21, *p*_3_ = 0.13 for d-LNMO, and *p*_1_ = 0.86, *p*_2_ = 0.14 for o-LNMO.

For d-LNMO, the multi-site model correctly describes the smeared-out chemical capacitance curve towards higher electrode potentials, although the calculated curve still exhibits a wavier profile than the experimental data. In principle, this could be corrected by assuming a larger number of 
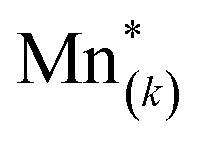
 sites with slightly different values of 
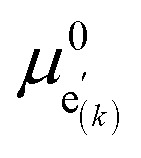
. Given the large variety of possible local chemical environments of oxygen vacancies in disordered LNMO,^17^ this would be a plausible assumption.

For o-LNMO, the multi-site model also gives a good fit of the experimental data, with the peak at 4.17 V being significantly diminished compared to d-LNMO and the third peak at 4.28 V being completely obsolete. Thus, while the *C*^V^_chem_ curve of d-LNMO in the oxygen vacancy regime is stretched out towards higher electrode potentials due to additional stabilisation of Mn*^3+^, the o-LNMO sample largely follows the single-site behaviour from Fig. 8b. This suggests that the two samples differ not only in their oxygen vacancy concentrations, but also in the variety of local chemical environments surrounding each oxygen vacancy, which excellently agrees with the well-established correlation of Ni-disorder and oxygen-deficiency.^17^

**Fig. 10:**
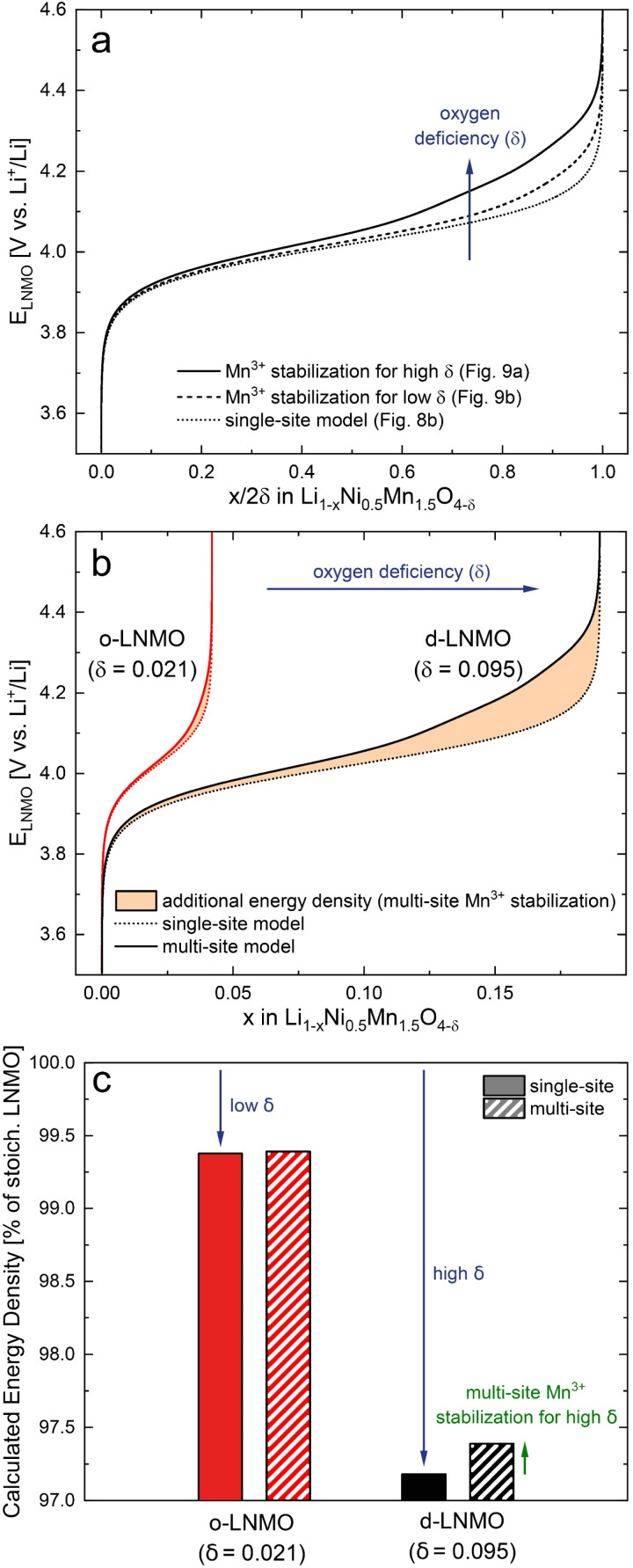
(a) Charge curves of the oxygen vacancy regime calculated by integration of *C*^V^_chem_*via*eqn (7) for the single-site model in Fig. 8b and the multi-site models in Fig. 9. Capacities were normalised by 2*δ* to allow a direct comparison between d-LNMO and o-LNMO. (b) Calculated charge curves of d-LNMO and o-LNMO showing the impact of oxygen deficiency and additional Mn*^3+^ stabilisation in the multi-site model. (c) Impact of oxygen deficiency and defect interactions on the calculated energy density of LNMO compared to stoichiometric LNMO for 0 ≤ *x* ≤ 1. For the Ni regime (2*δ* ≤ *x* ≤ 1), an average electrode potential of 4.72 V *vs.* Li^+^/Li was assumed.

Finally, we can illustrate the impact of oxygen vacancies on the shape of charge curves, which are obtained by integration of *C*^V^_chem_ according to eqn (7). First, we compare the capacity-normalised charge curves of the oxygen vacancy regime for d-LNMO, o-LNMO and the ideal defect model in Fig. 8b. As shown in Fig. 10a, the additional stabilising defect interactions of oxygen vacancies and Mn*^3+^ in the multi-site model lead to an upwards shift of the charge curve towards higher electrode potentials, thereby raising the average voltage in the oxygen vacancy regime. As expected from the respective distributions of *C*^V^_chem_ in Fig. 9, this effect is more pronounced for d-LNMO than for o-LNMO. The corresponding increase in energy density is marked in Fig. 10b as the area between the calculated charge curves with and without multi-site electron (Mn*^3+^) stabilisation. Not only is the additional stabilisation of Mn*^3+^ stronger for d-LNMO than for o-LNMO, but the total impact on the average voltage, and hence energy density, is further scaled up by the higher level of oxygen deficiency (*δ*) in d-LNMO.

On the other hand, an increase in *δ* also reduces the average voltage of the overall tetrahedral-site regime by moving part of its capacity from the high-voltage Ni to the lower-voltage Mn (oxygen vacancy) region. This leads to a trade-off between charge compensation by Mn*^3+^, which lowers the average voltage, and the additional multi-site stabilisation of Mn*^3+^ by oxygen vacancies, which again compensates at least part of the voltage loss. As shown in Fig. 10c, the lower energy density of the o-LNMO sample compared to ideal stoichiometric LNMO is still dominated by the level of charge compensation due to oxygen deficiency, and only a very small fraction of this loss is regained by multi-site electron stabilisation. For d-LNMO, on the other hand, a significant fraction of the energy density loss is compensated, thus mitigating the negative impact of oxygen vacancies on the average voltage.

These considerations raise the question whether oxygen vacancies and the accompanying electron (Mn^3+^) stabilisation might also increase the average voltage and energy density in LiMn_2_O_4−*δ*_ cathodes. In fact, Wang *et al.* recently reported differential capacity curves of oxygen-deficient LMO samples, which appear shifted by more than +50 mV with respect to nearly stoichiometric LMO,^50^ and are therefore consistent with our results for oxygen-deficient LNMO.

## Experimental

### Sample preparation

Thin films of SrRuO_3_ (SRO) and LiNi_0.5_Mn_1.5_O_4−*δ*_ (LNMO) were deposited onto the polished side of (100)-oriented SrTiO_3_ (STO) single crystal substrates of dimensions 10 × 10 × 0.5 mm^3^ (MaTecK, Germany) by means of radio-frequency magnetron sputtering in a custom-built deposition chamber (Huber Scientific, Austria), using commercial 2′′ targets obtained from Advanced Engineering Materials (China) and Loyaltarget (China), respectively. To ensure a good electrical contact around the edges to the backside, the substrate edges were roughened with fine sandpaper and sputter-coated with a 5/200 nm bilayer of Ti/Pt by means of DC sputtering (room temperature, Ar atmosphere, 0.7/2.0 Pa, 5 mA cm^−2^) prior to SRO/LNMO deposition. The nominal substrate temperature was determined *via* a power-temperature calibration of the heating stage using a polished (100)-oriented Y:ZrO_2_ single crystal (9.5 mol% Y_2_O_3_, CrysTec, Germany) and an optical pyrometer, assuming a surface emissivity of *ε* = 0.9. SRO was deposited in a gas mixture of Ar : O_2_ = 3 : 1 at a total pressure of 2.5 Pa, a nominal substrate temperature of 650 °C, and a power of 60 W, resulting in a film thickness of approximately 80 nm, as estimated by TEM. LNMO was deposited under oxygen atmosphere at a pressure of 2.5 Pa, a nominal substrate temperature of 550 °C, and a power of 60 W, resulting in a film thickness of approximately 70 nm, as estimated by TEM. For d-LNMO, the sample was cooled down at a rate of 30 °C min^−1^ immediately after deposition while maintaining an oxygen partial pressure of 2.5 Pa. For o-LNMO, the nominal substrate temperature was kept at 550 °C after deposition while gradually flooding the deposition chamber with oxygen over the course of 5 h. For this purpose, a separate temperature calibration at atmospheric pressure was used. Finally, the o-LNMO sample was kept at 550 °C under 1 atm of oxygen for 1 h, followed by a cool-down at 15 °C min^−1^. After LNMO deposition, the sample backside was sputter coated with another Ti/Pt bilayer to ensure a good electrical contact to the steel plunger of the electrochemical measuring cell. Due to the characteristic concentric variation of the deposition rate during magnetron sputtering and the large substrate area compared to the target size, a certain degree of thickness variation in the SRO and LNMO thin films across the sample is expected, and the film thicknesses measured by TEM can only be taken as approximate values for the whole sample.

### Structural characterisation

Out-of-plane X-ray diffractograms were recorded from 2*θ* = 15° to 80° on an Empyrean X-ray diffractometer (Malvern Panalytical, UK) using a hybrid *K*_α_ monochromator of type 2XGe(220) on the incident beam side and a GaliPIX3D area detector in scanning line mode on the diffracted beam side. AFM images of the sample surface were acquired on a Nanoscope V multimode setup (Bruker) and analysed using the open-source software Gwyddion.^51^ Electron-transparent lamellae for TEM investigations were prepared by standard lift-out techniques on a Thermo Fisher Scios 2 DualBeam FIB/SEM operating with a Ga-ion beam at 30 kV accelerating voltage. Final thinning and low-voltage polishing steps were carried out at 5 kV and 2 kV to reduce the amount of surface amorphization on the lamellae. All TEM imaging was carried out on a JEOL JEM-2100F field-emission gun microscope equipped with an image-side spherical aberration corrector, operating at an accelerating voltage of 200 kV. TEM images were acquired on a Gatan Orris SC1000 CCD camera. High-resolution TEM images were further processed using an average background subtraction filter (ABSF).

### Electrochemical characterisation

For electrochemical measurements, the thin-film samples were assembled in a two-electrode cell (PAT-Cell, EL-CELL, Germany) using a 260 μm glass-fibre separator (EL-CELL), 80 μL of a standard organic liquid electrolyte (1 M LiPF6 in a 1 : 1 mixture of ethylene carbonate and dimethyl carbonate, Aldrich, USA), and a Li metal counter electrode (10 × 10 × 0.6 mm^3^, Goodfellow, Germany). All electrochemical measurements were carried out at room temperature on a SP200 Biologic potentiostat with a built-in impedance analyser. Cyclic voltammetry was carried out with a scan rate of 1 mV s^−1^ in the voltage range of 3.7 to 4.9 V *versus* Li^+^/Li for 5 cycles before starting the series of impedance measurements. Potential-controlled impedance spectra were acquired in the frequency range of 200 kHz to 10 mHz (6 points per decade) using a perturbation amplitude of 10 mV. In the voltage range of 3.8 to 4.9 V *versus* Li^+^/Li, spectra were recorded in increments of 10 mV with intermittent equilibration (*i.e.*, constant voltage) steps of 5 min. The sufficiency of this equilibration time was verified by monitoring the flattening out of the exponential current decrease in response to an applied voltage step and comparing impedance spectra measured after various equilibration times. Since the measured current decreases to virtually static levels within 5 min and no significant changes of the impedance spectra are observed for longer equilibration times, we regard this time as sufficient to reach equilibrium.

## Conclusions

The electrochemical properties (charge transfer resistance *R*_ct_, ionic conductivity *σ*_ion_, specific chemical capacitance *C*^V^_chem_ and chemical diffusion coefficient *D̃*) of LiNi_0.5_Mn_1.5_O_4−*δ*_ thin films were investigated by cyclic voltammetry and impedance spectroscopy as a function of SOC for high (d-LNMO) and low (o-LNMO) levels of oxygen deficiency *δ*. The extracted properties vary by up to three orders of magnitude, with the most notable differences between d-LNMO and o-LNMO being observed in the oxygen vacancy regime below approximately 4.3 V *versus* Li^+^/Li, where d-LNMO exhibits significantly higher values of 1/*R*_ct_, *σ*_ion_, *C*^V^_chem_, and (up to 4.15 V) *D̃* than o-LNMO. Overall, the measured electrochemical properties are in excellent agreement with a defect chemical model based on ionic and electronic lattice site restrictions, with oxygen vacancies acting as a donor dopant.

Closer analysis of the oxygen vacancy regime revealed that oxygen vacancies are not merely charge-compensated by Mn^3+^ or Li vacancies, but are involved in defect interactions that significantly impact the charge curve. For both d-LNMO and o-LNMO, the charge curve below 4.0 V is accurately described by a single-site defect model that treats the tetrahedral Li sites T* and Mn sites (Mn*) as locally restricted to the proximity of an oxygen vacancy due to stabilisation (*i.e.*, trapping) of electrons and Li vacancies. For a high level of oxygen deficiency (d-LNMO), the charge curve in the oxygen vacancy regime bends upwards towards higher electrode potentials, thus mitigating the voltage suppression introduced through to the presence of Mn*^3+^. This characteristic feature of the charge curve suggests that the oxygen vacancy concentration not only affects the amount of redox active Mn*^3+/4+^, but also causes multiple energetically different electron sites in the vicinity of an oxygen vacancy.

Overall, these results reveal the complexity of effects introduced by oxygen vacancies in oxide-based Li insertion materials, but also the power of chemical capacitance measurements to understand and interpret the corresponding phenomena. Moreover, this study once more highlights the relevance of defect chemical concepts for understanding the complex interplay of ionic and electronic charge carriers in all battery materials.

## Author contributions

A. E. Bumberger: conceptualization (equal), investigation (lead), writing – original draft, S. Raznjevic: investigation (supporting), Z. Zhang: funding acquisition, M. Kubicek: funding acquisition, G. Friedbacher: investigation (supporting), J. Fleig: supervision, conceptualization (equal), writing – review & editing.

## Conflicts of interest

There are no conflicts to declare.

## Supplementary Material

TA-011-D3TA05086F-s001
