# Supplementary material for: Chemical capacitance measurements reveal the impact of oxygen vacancies on the charge curve of LiNi0.5Mn1.5O4−δ thin films
Source: J Mater Chem A Mater. 2023 Oct 16;11(44):24072–88. doi: 10.1039/d3ta05086f (PMC10644792; doi:10.1039/d3ta05086f)
Supplement: TA-011-D3TA05086F-s001 [file TA-011-D3TA05086F-s001.pdf]

Electronic Supplementary Material (ESI) for Journal of Materials Chemistry A.
This journal is © The Royal Society of Chemistry 2023

Supporting Information

Chemical capacitance measurements reveal the impact of oxygen vacancies on the charge curve of $LiNi_{0.5}Mn_{1.5}O_{4-\delta}$ thin films

Andreas E. Bumberger,*[a] Sergej Raznjevic,[b] Zaoli Zhang,[b] Markus Kubicek,[a] Gernot Friedbacher, [a] and Juergen Fleig [a]

[a] *Institute of Chemical Technologies and Analytics, TU Wien, Vienna, Austria*

[b] *Erich Schmid Institute for Materials Science, Leoben, Austria*

* corresponding author

S1. Fourier Transform of HR-TEM Images

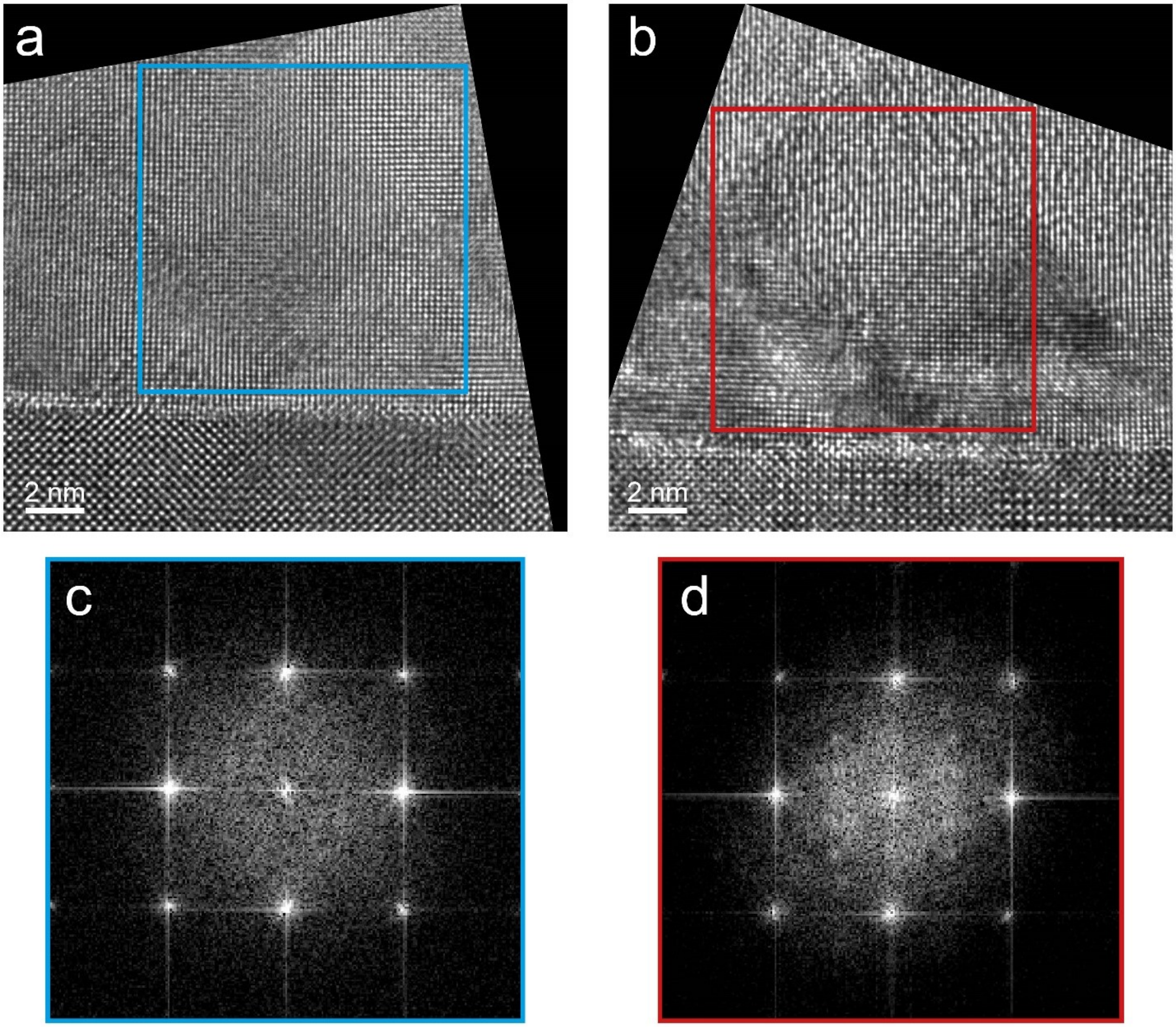

Fig. S1 (a-b) HR-TEM images of the d-LNMO (a) and o-LNMO (b) thin films on SRO (100) with colored squares indicating the region used for Fourier transform (FT) extraction. (c-d) FT-images of the d-LNMO (c) and o-LNMO (d) thin films extracted from the regions indicated in (a) and (b), respectively.

S2. Fitting of Impedance Spectra

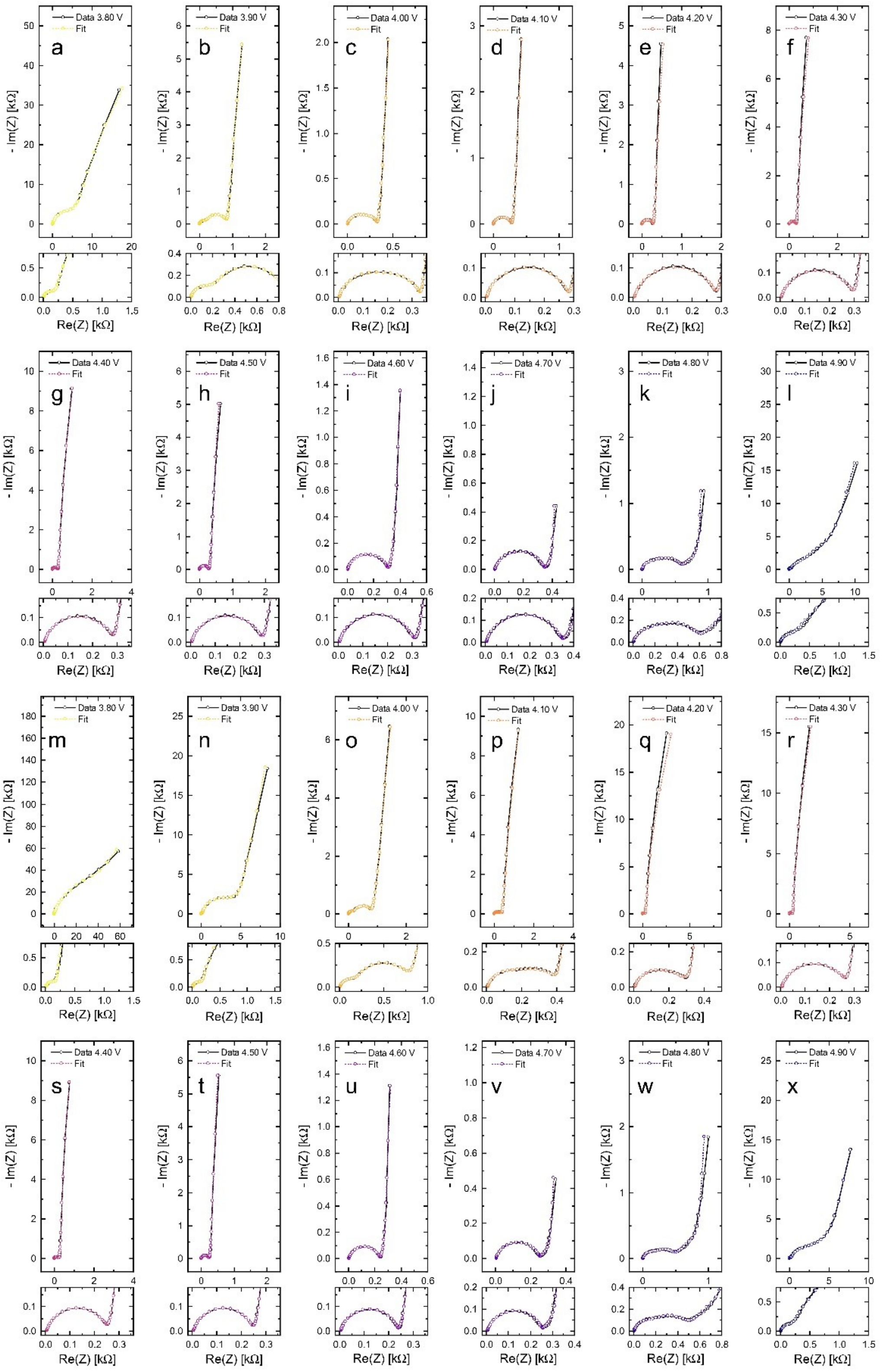

Fig. S2 Fits of all impedance spectra shown in Fig. 3 of the main manuscript. (a-l) d-LNMO (Fig. 3a). (m-x) o-LNMO (Fig. 3b).

S3. Total Electrode Resistance

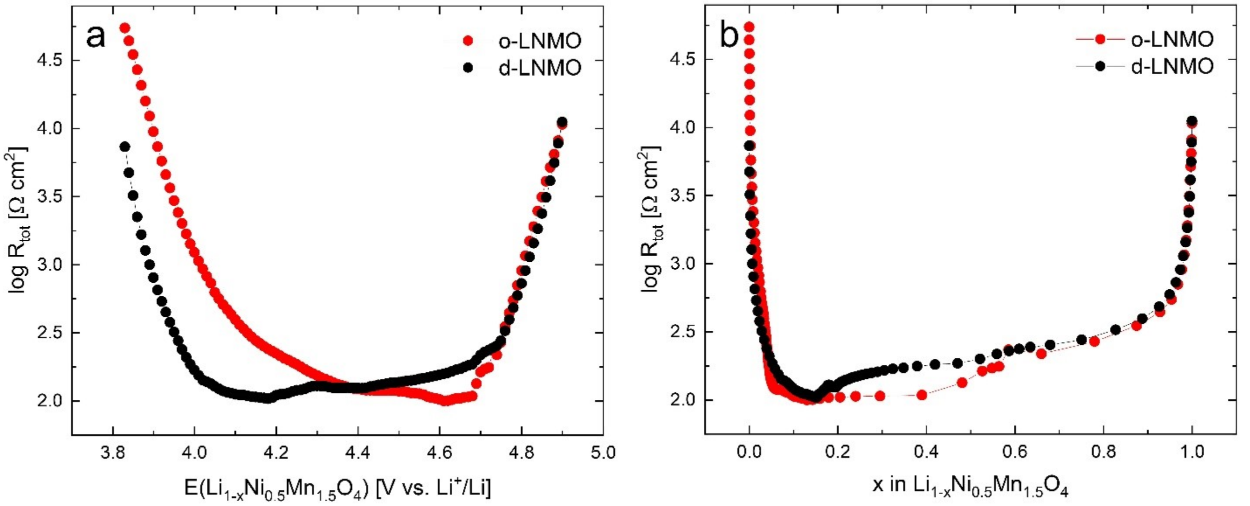

Fig. S3 Total effective electrode resistance $R_{tot} = R_{ion}/3 + R_{ct}$ as a function of electrode potential (a) and Li content (b). The Li content x was derived by normalization to the total electrode capacity obtained from the integration of C_{chem}^{V} via eqn (7) of the main manuscript.

S4. Full Defect Chemical Models

S4.1. Stoichiometric LNMO

Here we derive the general form of the Li chemical potential, and hence the chemical capacitance, for the case of multiple Li sites with different vacancy formation energies (e.g., octahedral and tetrahedral Li sites in a spinel structure) and multiple redox couples with different redox potentials (e.g., Mn and Ni in $Li_{1-x}Ni_{0.5}Mn_{1.5}O_{4-\delta}$). We start by considering the general insertion equilibrium of Li into an arbitrary material according to

$$Li + V_{Li(i)}^{'} \rightleftharpoons e_{(k)}^{'} \qquad (S1)$$

where Li corresponds to formally neutral lithium in the external phase (i.e., a Li ion from the electrolyte together with an electron from the current collector according to $Li = Li_{yte}^{+} + e_{cc}^{'}$) that defines the Li chemical potential with which the material is in equilibrium. $V_{Li(i)}^{'}$ and $e_{(k)}^{'}$, in Kröger-Vink notation, correspond to a Li vacancy at lattice site i and an electron at transition metal site k. For example, in LNMO, $e_{(Mn)}^{'}$ corresponds to Mn^{3+} and $e_{(Ni)}^{'}$ corresponds to either Ni^{3+} or $\frac{1}{2}Ni^{2+}$. As discussed in the main manuscript, we treat Ni as a single type of redox couple with a capacity of one electron per formula unit, hence we stick to the label $e_{(Ni)}^{'}$ without specifying its atomistic meaning. For further calculations, it is useful to reference the concentration $[j]$ of species j to the total concentration of formula units c^0 according to

$$x_j = \frac{[j]}{y_j \cdot c^0} \qquad (S2)$$

with x_j being the site occupancy of species j and y_j the corresponding number of sites per formula unit. For example, $y_T = y_O = 1$ for the tetrahedral (T) and octahedral (O) Li sites in a typical spinel material. In the case of LNMO, two different transition metals (Mn and Ni) act as active redox couples in the insertion reaction with corresponding capacities of $y_{Mn} = 1.5$ and $y_{Ni} = 1$. The goal is now to obtain (i) an expression for the total Li chemical potential μ_{Li} and (ii) the concentration of all electronic and ionic species as a function of μ_{Li}, i.e., a Brouwer diagram. Although the latter could be obtained by solving the corresponding system of mass action laws combined with the appropriate charge neutrality equations, it is not straightforward to obtain an expression for μ_{Li}, and hence the chemical capacitance, via this approach. As in our previous study on on $LiMn_2O_4$,[1] we therefore approach the defect chemical calculation via the separate chemical potentials of ionic and electronic charge carriers, as shown in the following.

For the insertion equilibrium in eqn (S1) we can formulate the corresponding balance of chemical potentials as

$$\mu_{Li} + \mu_{V_{Li}'} = \mu_{e'} \qquad (S3)$$

with μ_{Li} being the total Li chemical potential. We note that equilibrium conditions require that all Li sites share a common Li vacancy chemical potential $\mu_{V_{Li}'}$ and all electrons share a common electron chemical potential $\mu_{e'}$, which can be written as

$$\mu_{V_{Li}'} = \mu_{V_{Li(i)}'} = \mu^0_{V_{Li(i)}'} + kT\ln\frac{x_{V_{Li(i)}'}}{1 - x_{V_{Li(i)}'}} \qquad (S4)$$

and

$$\mu_{e'} = \mu_{e_{(k)}'} = \mu^0_{e_{(k)}'} + kT\ln\frac{x_{e_{(k)}'}}{1 - x_{e_{(k)}'}} \qquad (S5)$$

As described in our previous study on $LiMn_2O_4$,[1] it is convenient to rearrange eqns (S4) and (S5) to obtain the individual site fractions $\theta_{V_{Li(i)}^{'}}$ and $\theta_{e_{(k)}^{'}}$ according to

$$\theta_{V_{Li(i)}^{'}} = \frac{\left[V_{Li(i)}^{'}\right]}{c^0} = \frac{y_{V_{Li(i)}^{'}}}{1 + e^{\frac{\mu_{V_{Li(i)}^{'}}^{0} - \mu_{V_{Li}^{'}}}{kT}}} \qquad (S6)$$

and

$$\theta_{e_{(k)}^{'}} = \frac{\left[e_{(k)}^{'}\right]}{c^0} = \frac{y_{e_{(k)}^{'}}}{1 + e^{\frac{\mu_{e_{(k)}^{'}}^{0} - \mu_{e^{'}}}{kT}}} \qquad (S7)$$

The total vacancy and electron site fractions can then be expressed as

$$\theta_{V_{Li}^{'}} = \sum \theta_{V_{Li(i)}^{'}}(\mu_{V_{Li}^{'}}) \qquad (S8)$$

and

$$\theta_{e^{'}} = \sum \theta_{e_{(k)}^{'}}(\mu_{e^{'}}) + c \qquad (S9)$$

An additional correction term c was introduced in eqn (S9(to describe a possible shift of $\theta_{e^{'}}$ with respect to $\theta_{V_{Li}^{'}}$, as discussed below. The value of c can be derived by comparing the values of $\theta_{e^{'}}$ and $\theta_{V_{Li}^{'}}$ at the compositional boundaries of the material. For example, for stoichiometric LNMO with $y_T = y_O = 1$, $y_{Mn} = 1.5$, and $y_{Ni} = 1$, the total site fractions $\theta_{V_{Li}^{'}}$ and $\theta_{e^{'}}$ should vary from 0 to 2 and 2 to 0, respectively, as the material is oxidized from $Li_2Ni_{0.5}^{2+}Mn_{1.0}^{3+}Mn_{0.5}^{4+}O_4$ to $Ni_{0.5}^{4+}Mn_{1.5}^{4+}O_4$. This requires a value of $c = 0.5$ to account for the fact that in the fully reduced state of the material, i.e., at full occupancy of the octahedral

sites, 0.5 formula units of Mn^{4+} remain. The correction term c can also be conveniently used to describe the effect of dopants, as described in the next section.

Finally, the total chemical potentials $\mu_{V_{Li}'}(\theta_{V_{Li}'})$ and $\mu_{e'}(\theta_{e'})$ of Li vacancies and electrons, respectively, can be obtained by numerical inversion of eqns (S8) and (S9) and inserted into eqn (S3) to obtain the total Li chemical potential, and hence the chemical capacitance via eqn (1) of the main manuscript. To obtain the concentration of all relevant species as a function of Li chemical potential, $\mu_{V_{Li}'}(\theta_{V_{Li}'})$ and $\mu_{e'}(\theta_{e'})$ can simply be inserted into eqns (S6) and (S7). The concentrations of species that are not explicitly considered in the above calculation, i.e. Mn^{4+}, $Li_{(O)}^{+}$ and $Li_{(T)}^{+}$ can be obtained via

$$x_{Mn^{4+}} = 1 - x_{Mn^{3+}} \qquad (S10)$$

and

$$x_{Li_{(i)}^{+}} = 1 - x_{V_{Li(i)}'} \qquad (S11)$$

The resulting Brouwer diagram for stoichiometric LNMO, including the corresponding chemical capacitance, is shown in Figure 7a of the main manuscript. The values $\mu_{V_{Li(O)}'}^{0}/q = 2.90\,V$, $\mu_{V_{Li(T)}'}^{0}/q = 4.02\ V$, $\mu_{V_{Li(Mn)}'}^{0}/q = 0.00\,V$, and $\mu_{V_{Li(Ni)}'}^{0}/q = 0.70\,V$ (q = elementary charge) were chosen such that the chemical capacitance peaks (i.e., charge curve plateaus) occur around the same electrode potentials as observed experimentally.

S4.2. Oxygen-deficient LNMO

The defect chemical calculation of stoichiometric LNMO can easily be adapted for the presence of an electronically compensated donor dopant, such as oxygen vacancies, by subtracting the donor site fraction δ from the correction term c according to

$$c = 0.5 - z \cdot \delta = 0.5 - \frac{2 \cdot \left[V_O^{\bullet\bullet}\right]}{c^0} \qquad (S12)$$

where z is the charge number of the corresponding donor species. For oxygen vacancies $V_O^{\bullet\bullet}$, $z = 2$. The adapted Brouwer diagram for oxygen-deficient LNMO is shown in Figure 7b of the main manuscript for $\delta = 0.1$, with all other parameters remaining the same.

References

1. A. E. Bumberger, C. Boehme, J. S. Ring, S. Raznjevic, Z. L. Zhang, M. Kubicek and J. Fleig, *Chem Mater*, 2023, DOI: 10.1021/acs.chemmater.3c00814.
